# Pediatric Genetic Dystonias: Current Diagnostic Approaches and Treatment Options

**DOI:** 10.3390/life15070992

**Published:** 2025-06-20

**Authors:** Graziana Ceraolo, Giulia Spoto, Carla Consoli, Elena Modafferi, Gabriella Di Rosa, Antonio Gennaro Nicotera

**Affiliations:** 1 Unit of Child Neurology and Psychiatry, Department of Human Pathology of the Adult and Developmental Age “Gaetano Barresi”, University of Messina, 98125 Messina, Italy; graziana.c23@hotmail.it (G.C.); carlaconsoli@hotmail.com (C.C.); modafferielena91@gmail.com (E.M.); 2 Unit of Child Neurology and Psychiatry, Department of Biomedical Sciences, Dental Sciences & Morpho-Functional Imaging, University of Messina, 98125 Messina, Italy; giulia.spoto27@gmail.com; 3 Unit of Child Neurology and Psychiatry, Maternal-Infantile Department, University of Messina, 98125 Messina, Italy; antonionicotera@ymail.com

**Keywords:** pediatric dystonia, movement disorder, DYT1, chorea, dystonia–parkinsonism

## Abstract

Genetic dystonias are a heterogeneous group of movement disorders characterized by involuntary, sustained muscle contractions that cause repetitive movements and abnormal postures. Often beginning in childhood, they can significantly affect quality of life. Although individually rare, genetic causes are collectively relevant in pediatric dystonias, with over 250 associated genes. Among these, *TOR1A*, *SGCE*, and *KMT2B* are the most frequently reported in pediatric forms. Diagnosis is challenging due to the wide clinical and genetic variability. Recent advances in genetic testing, including whole-exome and whole-genome sequencing, have improved the early identification of causative variants. Functional data on selected mutations are helping to refine genotype–phenotype correlations. Management typically requires a multidisciplinary approach. Symptomatic treatments include anticholinergics, benzodiazepines, and botulinum toxin, while deep brain stimulation can be effective in refractory cases, especially in patients with *TOR1A* variants. Disease-modifying therapies are also emerging, such as gene therapy for AADC deficiency, highlighting the potential of precision medicine. This review provides an updated overview of pediatric genetic dystonias, with a focus on differential diagnosis and treatment strategies. Early and accurate diagnosis, together with personalized care, is key to improving outcomes in affected children.

## 1. Introduction

The term dystonia was first coined by Oppenheim in 1911 as “dystonia musculorum deformans”, referring to “muscle tone (that) was hypotonic at one occasion and in tonic muscle spasm at another, usually, but not exclusively, elicited upon voluntary movements” [1]. Since then, several attempts have been made to define and classify dystonia [2,3,4].

The term now encompasses both a motor phenomenon—characterized by sustained, twisting movements—and a clinical syndrome that may occur in isolation or be accompanied by other neurological or systemic features [5]. According to the current definition, dystonia is a movement disorder characterized by sustained or intermittent muscle contractions causing abnormal, often repetitive, movements, postures, or both [1].

Dystonia is often action-induced and influenced by specific tasks. Overflow (involuntary activation in distant muscles) and mirror dystonia (induced by contralateral voluntary actions) can intensify symptoms, while sensory tricks (*gestes antagonistes*) may reduce them [4,5]. Emotional states such as stress or anxiety may worsen dystonia, while sleep and rest typically bring relief [5].

The current classification of dystonia identifies two axes: (1) clinical characteristics, which distinguish dystonia according to age of onset, body distribution, temporal pattern, and associated clinical features; and (2) etiology, including identifiable anatomical changes in the nervous system detected through imaging or diagnostic testing, and patterns of inheritance [1].

Accordingly, dystonia may have an onset during infancy (birth to 2 years), childhood (3–12 years), adolescence (13–20 years), early adulthood (21–40 years), or late adulthood (>40 years). Moreover, it can involve a single body region (focal) or multiple regions, either in a contiguous (segmental) or non-contiguous (multifocal) manner; generalized dystonia involves the trunk and at least two other regions, with or without leg involvement, while hemidystonia is restricted to one body side, typically induced by acquired contralateral brain lesions [1].

The temporal pattern recognizes the static or progressive course of disease, also identifying if the dystonia is persistent, paroxysmal, action-specific, or has diurnal fluctuations. Clinically, dystonia may be categorized as “isolated” in the absence of additional motor signs, “combined” when co-occurring with other movement disorders, or “complex” when associated with broader systemic or neurological abnormalities [1,6]. Moreover, some authors proposed that an isolated tremor may itself constitute a dystonic manifestation [4]. According to the second axis of classification, dystonia can further be delineated as inherited (autosomal dominant or recessive, X-linked, and mitochondrial), acquired (due to structural or metabolic etiologies), or idiopathic [1].

**Figure 1:**
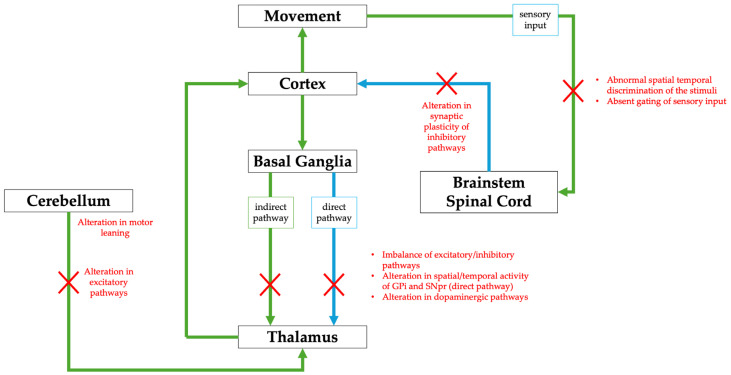
Simplified diagram of the pathophysiological mechanisms underlying dystonia. The motor cortex controls voluntary movement and receives excitatory and inhibitory inputs from the thalamus, cerebellum, brainstem, and spinal cord. The basal ganglia play a central regulatory role in movement initiation through opposing pathways: the direct pathway promotes motor activity, while the indirect pathway suppresses it. An imbalance between excitatory and inhibitory signals within these circuits has been proposed as a key mechanism underlying dystonia. In addition, alterations in dopaminergic pathways have been shown to impact this balance, as evidenced by genetic variants affecting dopamine signaling and the clinical response observed in dopa-responsive dystonias. More recent evidence suggests that abnormal spatial and temporal activity within the direct pathway, particularly involving the globus pallidus internus (GPi) and substantia nigra pars reticulata (SNpr), may contribute to dystonia pathophysiology. The cerebellum has also been implicated, with disruptions in the cerebello–thalamo–cortical pathway and deficits in motor learning processes. Altered spatial and temporal discrimination of sensory stimuli appears to further contribute to dystonia onset, as highlighted by the presence of sensory tricks that temporarily relieve symptoms. This supports the hypothesis of a defective sensory gating mechanism, which normally regulates and adjusts motor responses to peripheral sensory inputs, enabling appropriate compensatory movements. Finally, disrupted neural plasticity, especially within inhibitory circuits descending from the brainstem and spinal cord, has been suggested as an additional mechanism contributing to the development of dystonia. Excitatory pathways are shown in green, inhibitory pathways in blue, and alterations leading to dystonia in red [11].

Although dystonia is identified as a hyperkinetic movement disorder, it may also exhibit hypokinetic features at the same time [7]. In fact, the recent literature suggested that dystonia arises from a disorganization of the anatomical network encompassing extensive motor and sensory brain regions, which could be derived from structural lesions or genetic disorders [8,9]. Although dystonias were traditionally considered as disorders of the basal ganglia, recent evidence revealed the involvement of different structures, such as the cerebellum, thalamus, brainstem, spinal cord, and other cortical regions, supporting the concept that they represent, on the contrary, a network disorder caused by abnormalities in the nodes of the network or the functional connectivity between the nodes (See Figure 1) [10,11].

In this light, dystonia can be viewed as a neurodevelopmental circuit disorder, particularly in children, who usually show an evolving pattern [12,13,14,15]. For instance, congenital hypotonia may precede the onset of movement symptoms, which evolve with brain maturation, even in non-progressive conditions [14,16].

Traditionally, childhood-onset dystonias are more frequently linked to genetic or metabolic causes compared to adult forms. Moreover, in children, ongoing brain maturation and high neuroplasticity often lead to a relatively more benign disease course [17].

Dystonia is one of the most common hyperkinetic movement disorders in the pediatric population, and the prevalence of genetic forms has been estimated at 16.43/100,000 [18,19]. Initially named using chromosomal locus symbols (i.e., DYT1), Marras et al. proposed a new revised system that favors the use of the name of the gene responsible for the disorder, preceded by the prefix indicating the predominantly associated movement disorder (i.e., DYT for dystonia, PARK for parkinsonism, CHOR for chorea, NBIA for neurodegeneration with brain iron accumulation, and HSP for hereditary spastic paraplegia) [20]. More than 250 genes have been associated with dystonia, but their contribution to its symptomatology remains poorly defined, making a genotype–phenotype correlation challenging due to overlapping clinical features [14,21]. However, identifying the genetic etiology of dystonia is fundamental, as it can aid in selecting treatment options, such as levodopa for dopa-responsive dystonia, copper-chelating agents for Wilson’s disease (WD), or low-dose antiseizure medications for paroxysmal kinesigenic dyskinesia (PKD), while also potentially altering the course of the disease, such as, for instance, through gene therapy [14,22]. A thorough clinical evaluation, including patient history, physical examination, neuroimaging, and electrophysiological studies, is crucial for accurate diagnosis.

In this review, we aim to provide a comprehensive overview of pediatric genetic dystonias, highlighting the current understanding of their clinical classification, underlying genetic mechanisms, and pathophysiology. According to a clinical approach, we discussed the various forms of dystonia, starting with the most frequent forms and grouping them according to their clinical presentation and the molecular pathways underlying the disease. We also discussed the diagnostic approach, including the role of neuroimaging and genetic testing, as well as the available pharmacological and non-pharmacological treatment strategies. By integrating recent advances in the field, we sought to offer insights into the challenges and future directions in the diagnosis and management of these complex movement disorders.

## 2. Methods

We conducted an extensive review of the literature focusing on genetic dystonias in the pediatric population. Our research included the PubMed database, where we identified relevant genes using search terms such as “genetic dystonia”, “childhood-onset dystonia”, and “pediatric dystonia”. The selected genes were then confirmed and further characterized using OMIM, GeneCards, MalaCards, and MDSGene. We included original research articles, clinical case series, and relevant reviews written in English involving pediatric populations, with particular attention to genotype–phenotype correlations, age of onset, clinical presentation, and diagnostic and treatment strategies. We excluded reviews and manuscripts reporting non-genetic causes of dystonia, as well as genes associated with dystonia phenotypes with an onset after 18 years of age, or lacking a clear clinical and genetic characterization.

## 3. Isolated Dystonias

This group encompasses the genetic forms characterized by predominant dystonic symptoms in the absence of identifiable central nervous system injury. To date, nine genes have been mainly related to isolated dystonias.

DYT-*TOR1A* is the most common genetic form of early-onset isolated dystonia (dystonia 1, DYT1, Early-Onset Torsion Dystonia, Oppenheim’s Dystonia, OMIM#128100). It is an autosomal dominant disease with a penetrance of approximately 30%. DYT-*TOR1A* typically begins in late childhood, with a median age of onset of 13 years [23]. In a minor percentage of individuals, dystonia can occur during adolescence or adulthood [24]. The global incidence is estimated at 1–2/100,000 individuals, but it is significantly higher in certain populations, such as Ashkenazi Jews, where it can reach up to 100/100,000 individuals [24,25]. *TOR1A*, located on chromosome 9q34, was the first gene identified as responsible for isolated forms of dystonia [24,25,26]. It encodes a protein called TorsinA, an adenosine triphosphatase located in the endoplasmic reticulum and involved in a variety of cellular functions, including protein folding, lipid metabolism, cytoskeletal organization, and nuclear polarity. The most common pathogenic variant is an in-frame deletion of a GAG triplet [24,25]. Dystonia typically begins in the lower limbs and progresses to involve axial musculature, generalizing in approximately 50% of cases. Laryngeal and cranio-cervical involvements are uncommon. Limbs and trunk involvement can be particularly disabling, often rendering patients unable to walk without assistance. Notably, an earlier age at onset is associated with a more severe clinical phenotype [13,27]. The penetrance of dystonia appears to be influenced by both genetic and environmental factors, contributing to the phenotypic variability of the disorder. Among the identified genetic modifiers, a single-nucleotide variant, p.(Asp216His), has been shown to act as a susceptibility factor, significantly reducing the penetrance of the GAG deletion [13,25].

DYT-*THAP1* (dystonia 6, DYT6, OMIM#602629) is an autosomal dominant disorder caused by heterozygous loss-of-function variants in *THAP1*, located on chromosome 8p21. This gene encodes a transcription factor known as thanatos-associated protein domain-containing apoptosis-associated protein 1 (THAP1) [13,25,27]. Although it typically manifests during adolescence or adulthood, cases with childhood onset have also been reported. It was first identified in three Amish–Mennonite families [28]. Several pathogenic variants have been described, including missense, frameshift, and nonsense variants. The disorder exhibits an estimated penetrance of approximately 60%, although the factors contributing to its reduced penetrance remain poorly understood [13,25]. This type of dystonia typically presents with a multifocal/segmental distribution. Unlike DYT1 dystonia, symptoms usually begin in the upper limbs and frequently progress to involve the neck, craniofacial, or laryngeal muscles. Although the symptom distribution varies widely, cranial involvement is common, often leading to significant disability due to speech difficulties accompanied by dysarthria and/or dysphonia [13,27]. Over time, this condition tends to follow a rostro-caudal pattern of progression and typically persists throughout life [25]. This particular distribution, combined with the age of onset, can help differentiate this form of dystonia from DYT1 during the early stages [29].

The pathogenic mechanisms underlying DYT1 and DYT6 share common molecular underpinnings. Notably, a significant functional link has been proposed between *THAP1* and *TOR1A*, as *THAP1* directly binds to the *TOR1A* promoter. The disruption of *THAP1* function impairs its regulatory control over *TOR1A*, potentially contributing to dystonia pathogenesis through a shared molecular pathway [24,30]. In addition, dysregulation of the integrated stress response has been observed in several monogenic forms of dystonia, including those associated with variants in *TOR1A*, *THAP1*, *PRKRA*, *EIF2AK2*, and *SGCE* genes, suggesting a converging pathogenic mechanism. In particular, impaired signaling of eukaryotic initiation factor 2 alpha (eIF2α), a key activator of the integrated stress response, has been implicated in the pathogenesis of dystonia. The identification of aberrant eIF2α signaling in certain forms of dystonia opens promising avenues for targeted therapeutic interventions [31,32].

DYT-*KMT2B*, DYT-*HPCA*, DYT-*ANO3*, and DYT-*TUBB4A* are classified as isolated forms, since dystonia represents the predominant clinical feature. However, in contrast to the previously described genetic forms, they exhibit a more heterogeneous phenotype, with some cases showing mixed movement disorders. DYT-*KMT2B* (dystonia 28, DYT28, OMIM#617284) is a rare form of autosomal dominant generalized dystonia, caused by heterozygous variants in *KMT2B*, located on chromosome 19p13.12. It encodes a specific lysine methyltransferase that catalyzes the transfer of a methyl group to the fourth lysine (K4) of histone H3 (H3K4) [33]. Pathogenic variants in *KMT2B* typically lead to loss of function of the methyltransferase, supporting haploinsufficiency as the primary disease mechanism [34]. The clinical phenotype associated with *KMT2B*-related dystonia shares significant overlap with the presentation of DYT-*TOR1A*. Symptoms typically begin in the first decade of life, often between the ages of 4 and 6 years, with focal onset in the lower limbs. Unlike DYT1, the dystonia progresses to a generalized form, with prominent involvement of the cervical and cranial regions [18,33]. This type of dystonia was recently described and is often accompanied by additional clinical features, including epilepsy, eye movement abnormalities, intellectual developmental disorder (IDD), microcephaly, short stature, and mild facial dysmorphisms. Apart from dystonia, a small percentage of individuals exhibit other movement disorders, including myoclonic-like jerks, primarily affecting the limbs, as well as choreic and ballistic movements [35].

DYT-*HPCA* (dystonia 2, DYT2, OMIM#224500) is a rare autosomal recessive generalized dystonia caused by biallelic variants in *HPCA*, located on chromosome 1p35. It encodes hippocalcin, a neuron-specific calcium-binding protein family, crucial for regulating the activity of voltage-gated calcium and potassium channels [36]. First identified in a consanguineous Sephardic Jewish family, DYT-*HPCA* typically manifests in childhood with a variable phenotype, ranging from isolated generalized dystonia to forms combined with neurodevelopmental delay, IDD, and infantile seizures. Clinical phenotypes can vary in severity and distribution [37,38]. Dystonia usually begins in the limbs or neck and progressively spreads to other body regions, such as the perioral, cervical, and upper limb areas. Recently, four individuals with childhood-onset choreo-dystonia carrying a novel homozygous nonsense pathogenic variant in *HPCA* have been described [39].

DYT-*ANO3* (dystonia 24, DYT24, OMIM#615034) is a rare autosomal dominant form of multifocal/segmental or generalized dystonia caused by heterozygous variants in *ANO3* on chromosome 11p14. *ANO3*, which is highly expressed in the striatum, amygdala, hippocampus, and neocortex, encodes the anoctamin-3 protein, which functions as a calcium-activated chloride channel. It may play an important role in signal transduction and modulation of neuronal excitability [40]. To date, only a few pediatric patients have been reported [41]. The age of onset shows two peaks: infancy/childhood (≤12 years) and adulthood (40–49 years) [42]. The clinical phenotype is heterogeneous: dystonia may present either in isolation or combined with other movement disorders, typically beginning with cranio-cervical involvement and often accompanied by tremor, myoclonic jerks, blepharospasm, and oromandibular or laryngeal dystonia. Disease progression is usually slow, but symptoms may become more generalized over time [43]. Psychiatric symptoms (anxiety and depression), pain, and sleep disturbances have also been described [44].

DYT-*TUBB4A* (dystonia 4, DYT4, OMIM# 128101), also known as “whispering dysphonia”, is a distinct form of dystonia caused by pathogenic variants in *TUBB4A*, a gene located on chromosome 19p13. *TUBB4A* encodes β-tubulin 4A, a fundamental component of microtubules that constitute the cytoskeleton and play a pivotal role in various cellular functions [45]. Pathogenic variants occurring in early childhood typically result in a complex neurological disorder, clinically classified as hypomyelination with atrophy of the basal ganglia and cerebellum syndrome. These patients exhibit “whispering dysphonia”, characterized by a progressive impairment of voluntary phonation, ultimately resulting in a whispered speech pattern. The dystonia typically presents in a focal form, initially affecting the laryngeal musculature, but may progress to a segmental or generalized form, extending to the neck, face, and limbs. Unlike many other genetic dystonias, DYT4 is a late-onset disorder, with clinical manifestations predominantly emerging between the second and fourth decades of life [45,46].

DYT-*GNAL* (dystonia 25, DYT25, OMIM#615073) is a rare form of isolated dystonia caused by pathogenic variants in *GNAL*, located on chromosome 18 (18p11.21). This gene encodes the α subunit of the stimulatory G protein (Gαolf), which is highly expressed in the striatum [11]. DYT-*GNAL* typically manifests in adulthood, with a broad spectrum of phenotypes and genetic variants. However, the age of onset can range from childhood to adulthood. The condition follows an autosomal dominant inheritance pattern with reduced penetrance, and pathogenic variants in *GNAL* are predominantly loss-of-function alleles. *GNAL* variants are most commonly associated with focal or segmental dystonia, often affecting the laryngeal and cranio-cervical regions and frequently accompanied by tremor. However, a pediatric case of myoclonus has also been described [47,48]. Generalized forms are uncommon and tend to occur more frequently in childhood-onset cases. To date, biallelic *GNAL* variants have been identified in a limited number of cases, where a more complex phenotype has emerged. In these cases, generalized dystonia may co-occur with dysmorphic features, sensorineural hearing loss, and IDD [48,49]. Data from the literature have reported partial response to levodopa, possibly due to the modulation of dopamine signaling pathways. In fact, *GNAL* forms a heterotrimeric complex with G protein βγ subunits, playing a key role in dopamine signaling. This complex links dopamine D1 receptors to the activation of adenylyl cyclase type 5, leading to cyclic adenosine monophosphate (cAMP) production. The disruption of Gαolf impairs dopaminergic signal transduction in the striatum, potentially contributing to movement disorders [11].

Among the forms of generalized dystonia potentially associated with combined movement disorders, *VPS16* and *VPS41* are worthy of mention. These genes are located on chromosomes 20p13 and 7p14.1 and encode vacuolar protein sorting-associated proteins 16 and 41, respectively, both key components of the homotypic fusion and vacuole protein sorting (HOPS) complex [50]. Despite sharing a common molecular mechanism, *VPS16*- and *VPS41*-related disorders exhibit distinct clinical features.

*VPS41*-related disease (OMIM#619389) is caused by biallelic loss-of-function variants and is associated with a more severe phenotype, typically presenting in infancy, with profound neurodevelopmental impairment and progressive clinical deterioration during childhood [51]. Pathogenic variants in *VPS16* have been reported with both recessive and dominant inheritance patterns. Dystonia usually manifests in late childhood at a median age of 12 years [51,52].

The clinical phenotype of *VPS16*-related dystonia (OMIM#619291) is characterized by early-onset isolated dystonia, predominantly affecting the oromandibular, bulbar, cervical, and upper limb regions, followed by slow progressive generalization, typically preserving ambulation into adulthood. Neuropsychiatric features, including anxiety, depression, and emotional lability, as well as neurodevelopmental disorders, are also present [52,53].

*EIF2AK2* and *PRKRA*, located on chromosomes 2p22.2 and 2q31.2, respectively, have both been implicated among the monogenic causes of early-onset generalized dystonia, as they converge on the eIF2α phosphorylation pathway. While DYT-*PRKRA* (OMIM#612067) shows a primary combined dystonia phenotype and is, therefore, discussed in the following section, *EIF2AK2*-related disorder (OMIM#619687) typically presents as a predominantly isolated dystonia. *EIF2AK2* (eukaryotic translation initiation factor 2-alpha kinase 2), also known as protein kinase R, is one of the kinases activated as part of the integrated cellular stress response. It functions by phosphorylating eIF2α, thereby regulating protein synthesis under stress conditions. The activity of *EIF2AK2* is modulated by *PRKRA* (protein activator of the interferon-induced protein kinase), a double-stranded RNA-binding protein that acts as an essential co-activator of protein kinase R [31,54]. Dysfunction of the converging eIF2α pathway associated with these genetic variants has been linked to impairments in synaptic plasticity, which may be rescued through targeted modulation of eIF2α phosphorylation. Given the role of the eIF2α pathway in regulating neuronal long-term synaptic plasticity, *EIF2AK2* variants may represent a direct link between endoplasmic reticulum stress and aberrant synaptic plasticity, a well-established pathophysiological hallmark of dystonia [55]. *EIF2AK2* variants have been reported with both autosomal recessive and recessive inheritance patterns, and incomplete penetrance has also been described [31,54]. The clinical phenotype is broad and variable in severity. The most common features include childhood-onset generalized dystonia, with a median age at onset of 6 years. In more severe phenotypes, additional movement disorders such as parkinsonism may occur, along with developmental delay, language impairment, and seizures [56]. Dystonia commonly begins in the upper or lower limbs or the trunk and subsequently generalizes. An important diagnostic indicator is neurological deterioration following febrile illness or other physiological stress [31,54]. A potential risk factor for disease manifestation in individuals carrying genetic variants with reduced penetrance may be the response to viral infections, which can trigger the integrated stress response [32]. Functional studies on several disease-associated *EIF2AK2* variants have produced conflicting results, suggesting both gain-of-function and loss-of-function effects, depending on the specific variant [31].

The clinical features, neuroimaging findings, and treatment options of genetic isolated dystonias in the pediatric population are summarized in Table 1 [11,13,18,23,24,25,26,27,28,29,30,31,32,33,34,35,36,37,38,39,40,41,42,43,44,45,46,47,48,49,50,51,52,53,54,55,56].

## 4. Combined Dystonias

Combined dystonias refer to a group of disorders in which dystonia is the predominant movement symptom, occurring in combination with another movement disorder. Most commonly, dystonia is associated with parkinsonism and myoclonus, but other hyperkinetic disorders may also contribute to the clinical presentation. Table 2 reports the clinical features, neuroimaging findings, and available treatment options for genetically determined combined dystonias in the pediatric population [16,22,57,58,59,60,61,62,63,64,65,66,67,68,69,70,71,72,73,74,75,76,77,78,79,80,81,82,83,84,85,86,87,88,89,90,91,92,93,94,95,96,97,98,99,100,101,102,103,104,105,106,107,108,109,110,111,112,113,114,115,116,117,118,119,120,121].

### 4.1. Combined Dystonias with Parkinsonism

DYT/PARK-*GCH1* is a disease caused by pathogenic variants of *GCH1*, located in the 14q22.2 region. The gene encodes the GTP cyclohydrolase I, the rate-limiting enzyme in the synthesis of tetrahydrobiopterin, which is an essential cofactor for the three enzymes responsible for the production of tyrosine, serotonin, and L-dopa [56]. The autosomal dominant form of the disorder (dystonia 5, Segawa disease, DYT5a, DYT14, OMIM#128230) leads to reduced dopamine levels, resulting in a levodopa-responsive dystonia with diurnal fluctuations [58,59]. It is considered the most common inherited dystonia in children and adults, with a prevalence of 0.5 to 1.0 per million [60]. However, the condition primarily manifests in childhood and has variable penetrance, which is 4-fold greater in females [58,59]. DYT5a usually begins as a focal limb dystonia and slowly spreads rostrally, eventually generalizing during adolescence [22]. Other clinical presentations are a levodopa-responsive dystonia with parkinsonism that can occur with or without diurnal fluctuations, and an early-onset atypical dystonia that manifests in infancy and early childhood [61]. Moreover, GTP cyclohydrolase I deficiency can also follow an autosomal recessive inheritance pattern (OMIM#233910), with or without hyperphenylalaninemia, typically associated with a more complex presentation and more severe clinical manifestations, including truncal hypotonia, neonatal-onset rigidity, tremor, dystonia, spasticity, and oculogyric crises [59]. Three distinct phenotypes have been outlined: an early-infantile severe developmental and encephalopathic phenotype, an early-onset neurodevelopmental disorder with dystonia–parkinsonism, and a levodopa-responsive dystonia overlapping with the autosomal dominant form but with a wider age range of onset. While the first phenotype has been related mostly to homozygous pathogenic or likely pathogenic variants, milder phenotypes were reported in patients carrying compound heterozygous variants classified as pathogenic or likely pathogenic alongside variants of uncertain significance or likely benign [60]. Finally, an autosomal dominant form of paroxysmal exercise-induced dyskinesia (PED) has been described in a minority of patients [62].

DYT/PARK-*TH* (dystonia 5, Segawa syndrome, DYT5b, OMIM#605407) is an autosomal recessive dystonia, caused by biallelic variants in the *TH* gene, on chromosome 11 (11p15.5). It encodes the tyrosine hydroxylase, another essential and rate-limiting enzyme leading to the formation of dopamine and other catecholamines [63]. The clinical presentation ranges from a dopa-responsive infantile parkinsonism with a progressive hypokinetic–rigid syndrome to a severe progressive encephalopathy [64]. The former usually begins within 1 year of age (2 months to 5 years) with a focal leg dystonia that progressively involves the other leg, arms, trunk, face, and oropharyngeal musculature. Diurnal fluctuations or lasting for several days have been described in the early stages of the disease. Psychomotor development is normal or slightly delayed during the first 2–5 years of life, followed by a gradual motor deterioration that leads to wheelchair dependence within a few years [59]. The encephalopathy has a typical onset at birth, with generalized dystonia, tremor, myoclonus, and oculogyric crises [65]. A rapid progression leads to severe disability, intellectual impairment, and autonomic dysfunction [64].

DYT/PARK-*SPR* (OMIM#612716) is an autosomal recessive disease that results from biallelic pathogenic variants in the sepiapterin reductase gene, located on 2p13.2. Clinical onset occurs within the first year of life and is characterized by axial hypotonia, oculogyric crises, developmental delay, and dystonia [59]. DYT/PARK-*SPR* is typically action-induced [66]. Other neurologic and psychiatric features may be present, such as parkinsonism, tremors, dysarthria, distal hypertonia, IDD, psychiatric disorders, and sleep disturbances [59,66].

Dystonias related to pathogenic variants in the *GCH1*, *TH*, and *SPR* genes show a good response to levodopa treatment and, therefore, are defined as dopa-responsive dystonias. On the contrary, other forms of combined dystonia do not show an optimal response to levodopa treatment. This is the case for DYT-*PRKRA* (dystonia 16, DYT16, OMIM#612067), an autosomal recessive disorder caused by pathogenic variants in *PRKRA*, located on 2q31.2. The gene encodes a protein kinase that functions as an interferon-inducible activator of double-stranded RNA-dependent pathways. It was first identified in two consanguineous families by Camargos et al., who described patients with focal limb or cervical dystonia, with onset during the second decade of life. The dystonia showed a progressive course, leading to severe generalized dystonia, including opisthotonos, sardonic smile, and laryngeal involvement [67]. Although recessive inheritance has been confirmed in a Polish family [68], a single patient with a heterozygous variant in *PRKRA* has been reported [69].

DYT-*DDC* (aromatic L-amino acid decarboxylase deficiency, OMIM#608643) is a rare autosomal recessive disorder resulting from biallelic pathogenic variants in *DDC*, located on the 7p12.2-p12.1 region. This gene encodes the DOPA decarboxylase, an enzyme involved in the biosynthesis of the monoamine neurotransmitters dopamine, epinephrine, norepinephrine, and serotonin [70]. The phenotype is highly heterogeneous, but most patients have an onset within the first year of life and display symptoms such as hypotonia, movement disorders (i.e., dystonia, oculogyric crises, and hypokinesia), developmental delay, and autonomic symptoms. The outcome can vary from mild to severe motor and cognitive impairment associated with frequent behavioral and psychiatric conditions [70,71,72].

DYT/PARK-*ATP1A3* (dystonia 12, DYT12, rapid-onset dystonia–parkinsonism, OMIM#128235) is an autosomal dominant disorder caused by pathogenic variants in *ATP1A3*, located on 19q13.2. The gene encodes the alpha-3 catalytic subunit of the Na^+^/K^+^-ATPase transmembrane ion pump, and it is exclusively expressed in neurons, especially in the basal ganglia, hippocampus, and cerebellum [73]. The disease typically manifests during adolescence or early adulthood, with an acute or subacute onset of asymmetric dystonia often accompanied by mild parkinsonian features. Symptoms frequently appear following a triggering event, such as physical overexertion, trauma, heat exposure, or fever [74]. The course of disease is slowly progressive, following a rostro-caudal gradient, and showing a prominent bulbar dysfunction, including dysarthria and dysphagia [22]. Other abrupt exacerbations may occur throughout life [75]. Notably, *ATP1A3*-related disorders encompass various phenotypes, including alternating hemiplegia in childhood, CAPOS (cerebellar ataxia, peripheral neuropathy, optic atrophy, and sensorineural hearing loss) syndrome, and developmental and episodic encephalopathy, with overlapping clinical features, among these syndromes [22].

Infantile-onset parkinsonism–dystonia-1 (PKDYS1, OMIM# 613135) is an autosomal recessive disorder caused by biallelic disease-causing variants in *SLC6A3*, on chromosome 5 (5p15.33). The gene encodes a dopamine transporter that mediates the active reuptake of dopamine from the synapse and represents the principal regulator of dopaminergic neurotransmission [76]. The disease typically manifests within the first year of life. However, an atypical presentation with later onset in childhood, adolescence, or adulthood has also been described [77]. The classical phenotype is characterized by irritability, feeding difficulties, hypotonia, and delayed motor development. Over time, patients develop a hyperkinetic disorder featuring orolingual dyskinesia, dystonia, chorea, and ballism, which progressively evolves into dystonia–parkinsonism, eventually leading to akinesia. Episodic status dystonicus (SD) and oculogyric crises have also been documented as additional clinical features [78]. The late-onset form has been reported in a few individuals who exhibited attention deficit/hyperactivity disorder (ADHD) symptoms in childhood and later developed dystonia–parkinsonism and tremors, with onset occurring in childhood, adolescence, or adulthood [77].

Childhood-onset striatonigral degeneration (SNDC, OMIM#617054) is a very rare form of dystonia–parkinsonism. It is an autosomal recessive disorder that has been reported in children carrying biallelic variants in *VAC14*, located in the region 16q22.1–q22.2. This gene encodes a pentameric scaffold protein that regulates the synthesis of phosphatidylinositol 3,5-bisphosphate and, therefore, is implicated in the trafficking and acidification of the endolysosomes, autophagy, stress-induced signaling, and ion-channel activity [79]. The disease is characterized by the sudden onset of neurodegeneration with regression of developmental milestones during the first years of life. Patients develop impaired movement with dystonia, become nonverbal and nonambulatory, and show striatal abnormalities and increased accumulation of iron on brain imaging [80,81]. A favorable response to deep brain stimulation of the globus pallidus internus (GPi-DBS) has been reported and may be worth considering in those patients who do not respond to medical treatment [82].

Recently, autosomal recessive *WARS2*-related disorders have been reported. The gene is located on 1p12 and encodes the mitochondrial form of tryptophanyl-tRNA synthetase. Biallelic disease-causing variants have been associated with two prevalent phenotypes: a neurodevelopmental mitochondrial disorder with abnormal movements and lactic acidosis with or without seizures (NEMMLAS) and a childhood-onset movement disorder characterized by parkinsonism–dystonia (parkinsonism–dystonia 3, OMIM#619738) [83]. The last disorder has an onset usually within the first decade of life, with variable presentations ranging from focal limb dystonia or generalized hyperkinetic movements (ballistic and dystonic features) to parkinsonian symptoms (bradykinesia, rigidity, and tremors). Associated movement disorders, such as myoclonus and ataxia, have also been described. *WARS2*-related disorders showed a progressive course but an overall good response to dopaminergic agents, and in particular to levodopa, although some patients developed treatment-induced dyskinesia [83,84]. Interestingly, biallelic loss-of-function variants have been related to the epileptic phenotype, while all the patients displaying the movement disorder phenotype harbor the hypomorphic variant p.(Trp13Gly). This latter is relatively prevalent in the European population and slightly affects WARS2 protein function in the homozygous state [83]. In fact, Skorvanek and colleagues found that individuals carrying this variant often showed milder phenotypes and later disease onset. Nevertheless, the recurrence of this variant in multiple families with WARS2-related disease, along with its demonstrated deleterious effect in cellular assays, led the authors to propose a causative role for this allele in human disease when present *in trans* with more detrimental loss-of-function alleles [84].

A distinguishing feature in childhood-onset dystonia–parkinsonism syndromes may be the response to levodopa, which could help differentiate *GCH1*, *TH*, *SPR*, *DDC*, and *WARS2*-related disorders. Additionally, factors such as the age of onset, presence of diurnal fluctuation, the pattern of motor progression (i.e., focal to generalized dystonia in *GCH1* and *TH*), and involvement of bulbar muscles (as seen in *ATP1A3*-related disorders) can further aid in distinguishing between different genetic causes. The presence of oculogyric crises may also be useful for differentiating disorders related to the *TH*, *DDC*, and *SPR* genes, where they are more commonly observed.

### 4.2. Combined Dystonias with Myoclonus

Another group of pediatric combined dystonias with a genetic etiology may be associated with myoclonus as the predominant feature. Myoclonic dystonia, or myoclonus–dystonia, presents with dystonia as the core feature, but tremor or rapid, jerky movements resembling myoclonus may also be present [85].

DYT-*SGCE* (dystonia 11, DYT11, myoclonic dystonia, OMIM#159900) is an autosomal dominant disorder with reduced penetrance because of maternal imprinting [86]. It is caused by heterozygous disease-causing variants in the epsilon-sarcoglycan gene (*SGCE*) located on chromosomal region 7q21.3. It encodes single-pass transmembrane proteins that are part of the dystrophin–glycoprotein complex, and it is expressed in various brain regions, including the somatosensory and motor cortex, putamen, thalamus, hippocampus, and cerebellum [87]. The onset of the disease usually occurs during childhood, and it is characterized by myoclonus that increases in severity during action tasks (i.e., speaking, feeding, writing, or walking) and action dystonia during writing and walking, with a progression of motor symptoms [88]. A dramatic response to alcohol has been reported in adult patients [89,90].

DYT-*KCTD17* (myoclonic dystonia 26, DYT26, OMIM# 616398) is an autosomal dominant disorder characterized by the onset of myoclonic jerks in the upper limbs during the first or second decade of life. The disease follows a progressive course, with dystonia predominantly affecting the cranio-cervical regions and gradually spreading to the limbs, trunk, and oromandibular and laryngeal muscles [91,92]. *KCTD17* is located on 22q12.3 and encodes for a member of the potassium channel tetramerization domain (KCTD)-containing proteins [91]. *KCTD17* splice-site variants may be associated with a more severe presentation [93]. A good response to Gpi-DBS has been reported in several patients [91,92,93]. It has been proposed that patients presenting with *SGCE*-negative myoclonic dystonia should be tested for *KCTD17* [93].

Similarly, *KCNN2* (OMIM#619724) has been recently proposed as a gene responsible for *SGCE*-negative myoclonic dystonia. The gene is located on 5q22.3 and encodes a calcium-activated potassium channel. It was first reported in eight individuals over three generations of a single family, suggesting an autosomal dominant inheritance pattern [94]. Later, it was associated with a neurodevelopmental disorder with or without variable movement or behavioral abnormalities [95], while other groups reported de novo heterozygous variants of this gene in individuals affected by myoclonic dystonia [96,97,98]. The onset is typically in childhood and is often characterized by writing difficulties, resulting from focal dystonia and superimposed myoclonic jerks involving the upper limbs. However, there is some variability in the severity of symptoms, and the course has been described as non-progressive. Different from DYT-*SGCE*, this form of myoclonic dystonia presents with more distal myoclonus and cerebellar signs [94].

Recently, another form of myoclonic dystonia was described in patients carrying heterozygous variants in *GRIN2A* (OMIM#138253). The gene is located on 16p13.2 and encodes for the NR2 subunit of the N-methyl-D-aspartate (NMDA) receptor, a glutamate-activated ion channel permeable to sodium, potassium, and calcium that is found at excitatory synapses throughout the brain [99]. *GRIN2A* disease-causing variants have been previously associated with epilepsy–aphasia spectrum disorders, including Landau–Kleffner syndrome and epileptic encephalopathy with continuous spike-wave during slow-wave sleep, as well as atypical Rolandic epilepsy, autism spectrum disorder, and speech impairment. However, several reports in the literature also describe *GRIN2A* variants linked to non-epileptic neurodevelopmental delay and movement disorders [100,101,102]. Particularly, the phenotype is characterized by generalized early-onset dystonia, often associated with other movement disorders such as myoclonus, chorea, tremor, or ataxia, as well as other neuropsychiatric features (i.e., speech impairment, developmental delay, IDD, ADHD, and psychiatric disorders) [100,101,102,103].

The advent of advanced genetic techniques has led to the identification of an increasing number of variants potentially associated with disorders of previously unknown etiology, such as *SGCE*-negative myoclonic dystonia. As a result, additional genes have been proposed as potential contributors to this phenotype, although the current evidence remains sparse and primarily based on anecdotal observations. This is the case of the patient reported by Chawla and colleagues, who harbored a heterozygous likely pathogenic variant in *YY1* and showed a childhood-onset myoclonic dystonia involving the neck and upper limbs without cerebellar signs [104].

The causative role of *CACNA1B* (OMIM#601012) in the onset of myoclonus–dystonia was first proposed in a single family carrying the variant p.(Arg1389His) [105]. However, this association was subsequently refuted by a large multicenter study, which failed to confirm a clear link between the variant and the phenotype, thereby questioning its pathogenic role [106]. Although one additional pediatric case of myoclonic dystonia has been related to a *CACNA1B* variant in a Taiwanese large cohort of patients with isolated and combined dystonia [107], the variant carried by this particular patient is classified as likely benign (PM2, BP1, BP4) according to the American College of Medical Genetics and Genomics–Association for Molecular Pathology (ACMG-AMP) classification [108], leaving the role of *CACNA1B* in the onset of myoclonic dystonia still debatable.

A distinguishing feature in myoclonic dystonia syndromes is the response to specific triggers, such as the alcohol sensitivity in DYT-*SGCE*. DYT-*KCTD17* tends to present with upper limb myoclonus and cranio-cervical dystonia, while *KCNN2* is marked by more distal myoclonus and cerebellar signs. *GRIN2A* variants lead to early-onset generalized dystonia with additional neuropsychiatric symptoms. These clinical traits help differentiate the underlying genetic causes.

### 4.3. Combined Dystonias with Chorea

Some genes have been reported to be associated with a complex neurological phenotypic spectrum, including various hyperkinetic movement disorders such as dystonia, chorea, myoclonus, and dyskinesia [109]. Among these, *ADCY5* pathogenic variants have been related to dyskinesia with orofacial involvement, both in autosomal dominant and recessive forms (OMIM#606703 and #619647, respectively), and to a neurodevelopmental disorder with hyperkinetic movements and dyskinesia (OMIM#619651). The gene is located on the 3q21.1 region, and it encodes a striatal-specific enzyme that converts adenosine triphosphate (ATP) into cAMP, an intracellular second messenger crucial for several molecular pathways [110]. The clinical phenotype of CHOR/DYT-*ADCY5* is characterized by an early onset during infancy or childhood, with delayed milestones and axial hypotonia. Then, the patient usually develops a hyperkinetic movement disorder, mostly characterized by generalized chorea and dystonia with frequent exacerbations of dyskinesias upon awakening and when falling asleep [111,112]. Particularly, the nocturnal dyskinesia is a peculiar feature of this disease, allowing it to be distinguished from most other movement disorders, where dyskinesia disappears during sleep. Abnormal nocturnal movements usually occur during stage two and rapid eye movement sleep [109]. Interestingly, patients affected by heterozygous variants of *ADCY5* have shown a dramatic improvement after treatment with caffeine [113].

Similarly, the *GNAO1*-related phenotype encompasses a broad spectrum of clinical presentations, ranging from a developmental and epileptic encephalopathy (OMIM#615473) to a neurodevelopmental disorder with involuntary movements (OMIM#617493). The core features of the disease include early-onset epilepsy, developmental delay/IDD, and a hyperkinetic movement disorder such as chorea, dystonia, and myoclonus [114]. The gene is located on chromosome 16 (16q13) and encodes an alpha subunit of heterotrimeric guanine-nucleotide-binding protein G, which is regarded as the most abundant membrane protein in the mammalian central nervous system and represents about 1% of total brain membrane protein [115]. It acts as a modulator of cAMP and is involved in cytoskeletal remodeling and the functional polarity of developing neurons, thereby contributing to the regulation of synaptic function and neuronal excitability [116,117]. The disorder follows an autosomal dominant inheritance pattern, with onset during infancy or early childhood, with hypotonia and a regression of motor milestones in combination with epileptic symptomatology or severe dyskinesias with athetosis, chorea, and dystonia [118]. Notably, loss-of-function variants have been associated with a severe form of early infantile epileptic encephalopathy, while gain-of-function variants have been related to hyperkinetic disorders [16,119]. Dystonic storms or exacerbations of dyskinesia are a well-documented feature in these patients. However, a good response to DBS has been reported [118].

When addressing a patient presenting with a combination of dystonia and chorea, it is worth keeping in mind the *NKX2-1* gene. It is located on the 14q13 region and encodes a transcription factor expressed during early development of the thyroid, lungs, and brain. It is mainly responsible for rare childhood-onset chorea (OMIM#118700). However, some uncommon cases have been reported presenting with different movement disorders in association, such as chorea, dystonia, and ataxia [120,121].

In conclusion, *ADCY5* variants are characterized by nocturnal dyskinesia, which improves with caffeine, while *GNAO1*-related disorders usually present with dystonic storms. *NKX2-1* variants display childhood-onset chorea, which sometimes is associated with dystonia and ataxia, helping to differentiate these conditions.

## 5. Complex Dystonias

Complex dystonias are conditions in which dystonia is the predominant clinical feature within a broader phenotype that includes symptoms beyond movement disorders [122]. Particularly, dystonia distribution can be both generalized or focal and sometimes present the involvement of the tongue or perioral muscles (described as “risus sardonicus”). In addition, several neurologic and psychiatric conditions have been reported, including developmental delay, cognitive impairment, spasticity, ataxia, bulbar dysfunction (i.e., anarthria), visual and oculomotor disturbances, hearing loss, and seizures [123].

The list of complex dystonia forms is extensive and constantly evolving. In the following sections, we present a clinical classification of several complex dystonias, categorizing them according to the primary symptoms associated with the condition, such as spasticity, ataxia, parkinsonism, chorea, epilepsy, and hearing or ocular impairments.

### 5.1. Complex Dystonias in Combination with Spasticity

The coexistence of dystonia and spasticity is a common feature in several syndromes, each with distinct underlying pathophysiological mechanisms. In this section, we explored a range of these syndromes, including Aicardi–Goutières Syndrome (AGS) and NBIA, with a focus on their genetic basis and clinical manifestations. AGS is a genetic disorder caused by variants in several genes, such as *TREX1* (OMIM#225750), *RNASEH2A* (OMIM#610333), *RNASEH2B* (OMIM#610181), *RNASEH2C* (OMIM#610329), *SAMHD1* (OMIM#612952), *ADAR* (OMIM#615010), or *IFIH1* (OMIM#615846), and typically follows an autosomal recessive inheritance pattern [124,125]. AGS exhibits a broad phenotypic spectrum, ranging from severe neonatal-onset forms with profound functional impairment to milder late-onset forms. AGS commonly manifests as early-onset encephalopathy, which progressively leads to generalized dystonia, spasticity, seizure, and often, cognitive impairment [18,126].

NBIA is a heterogenous group of inherited neurological disorders characterized by abnormal iron deposition in the basal ganglia, commonly in the globus pallidus and/or substantia nigra [127]. NBIA arises from various genetic variants with different inheritance patterns, all leading to progressive neurodegeneration. Alongside spasticity and dystonia, clinical features may include dysarthria, neuropsychiatric abnormalities, parkinsonism, polyneuropathy, and visual impairment [6]. Among the NBIA subtypes, pantothenate kinase-associated neurodegeneration (PKAN, NBIA/DYT-*PANK2*, NBIA1, OMIM#234200), caused by disease-causing variants in the *PANK2* gene, is the most common [123]. A hallmark radiological feature is the “eye-of-the-tiger-sign” on T2-weighted images, reflecting focal iron accumulation in the globus pallidus, though this may disappear as the disease progresses [128]. PKAN presents in two clinical forms: the “classic/early” form, which typically manifests around 3 years of age with rapid progression and early gait abnormalities, and the “atypical” form, beginning after 10 years of age with a slower progression. Both are characterized by dysarthria, progressive dystonia, rigidity, spasticity, parkinsonism, hyperreflexia, retinal degeneration, neuropsychiatric abnormalities, and rarely, seizures [127]. Dystonia is the predominant extrapyramidal feature, particularly in the classic form, where it is often more pronounced in the limbs and face [129]. Other NBIA subtypes include mitochondrial membrane protein-associated neurodegeneration (MPAN, HSP/NBIA-*C19orf12*, NBIA4, OMIM#614298) and fatty acid hydroxylase-associated neurodegeneration (FAHN, HSP/NBIA-*FA2H*). MPAN, resulting from *C19orf12* variants, has a highly variable onset, ranging from early childhood to adulthood [130]. The initial symptoms often include gait difficulties, spasticity (mainly in the lower limbs), behavioral disturbances (such as emotional lability, anxiety, compulsions, depression, impulsiveness, and psychosis), cognitive decline, optic atrophy, motor axonal neuropathy, and bladder incontinence [131,132]. In early-onset cases, dystonia and spasticity are the predominant motor symptoms, while parkinsonism is more commonly seen in later-onset cases [130]. FAHN, caused by variants in *FA2H*, disrupts the function of fatty acid 2-hydroxylase, leading to early central nervous system involvement. Affected individuals commonly present with spasticity, ataxia, dystonia, and ocular abnormalities, such as optic atrophy and oculomotor dysfunction [133,134]. As the disease advances, progressive intellectual decline and seizures may emerge, while increasing spasticity and dystonia impair mobility, eventually resulting in wheelchair dependence [134].

Table 3 outlines the key genes identified in the literature as being associated with complex dystonia with spasticity in the pediatric population [6,18,124,125,126,127,128,129,130,131,132,133,134].

### 5.2. Complex Dystonias in Combination with Ataxia

A wide range of diseases can cause dystonia combined with ataxia in pediatric populations, including autosomal recessive cerebellar ataxias such as ataxia–telangiectasia, inborn errors of metabolism (IEMs), particularly the subgroup of lysosomal storage disorders (LSDs), such as Niemann–Pick disease type C (NPC), GM1 gangliosidosis, and fucosidosis, as well as neurodegenerative syndromes like *PLA2G6*-associated neurodegeneration and DYT-*SQSTM1* syndrome.

Ataxia–telangiectasia (OMIM#208900) is an autosomal recessive cerebellar ataxia linked to *ATM* gene variants, primarily characterized by cerebellar ataxia, oculocutaneous telangiectasia, and oculomotor apraxia. However, dystonia is a common feature, ranking as the second-most-frequent initial symptom after ataxia and the second-most-prevalent movement disorder after myoclonus [135,136].

Among LSDs, NPC is one of the most well-known conditions associated with movement disorders. It is an autosomal recessive neurovisceral LSD caused by disease-causing variants in the *NPC1* or *NPC2* genes (OMIM#607623 and #601015, respectively), leading to lipid accumulation in various organs, including the brain [6,137,138]. This results in cerebral degeneration, cognitive decline, psychiatric symptoms, and progressive neurological issues. Movement disorders, including cerebellar ataxia, generalized dystonia, and myoclonus, often present as early or prominent symptoms, alongside vertical supranuclear gaze palsy, dysarthria, and dysphagia [6,137,138,139]. In addition to NPC, other rare storage disorders, including GM1 gangliosidosis and fucosidosis, can also present with dystonia. GM1 gangliosidosis, caused by pathogenic variants in *GLB1*, leads to β-galactosidase deficiency, with its type III or adult form usually manifesting between 3 and 30 years of age. DYT/PARK-*GLB1* (OMIM#611458) is characterized by generalized dystonia, parkinsonism, pyramidal signs, dysarthria, cognitive deficits, skeletal abnormalities, short stature, corneal clouding, and cardiomyopathy [122,140,141,142]. Similarly, fucosidosis (OMIM#230000), a rare autosomal recessive LSD caused by variants in *FUCA1*, results in α-L-fucosidase deficiency [143]. It presents with progressive motor and cognitive decline, seizures, and systemic manifestations, such as coarse facial features, dysostosis multiplex, recurrent upper respiratory infections, angiokeratoma corporis diffusum, and visceromegaly [143,144]. Although focal or generalized dystonia is a recognized feature, it is rarely reported in fucosidosis [143,145,146,147].

*PLA2G6*-associated neurodegeneration encompasses three overlapping phenotypes: infantile neuroaxonal dystrophy (INAD) and atypical neuroaxonal dystrophy (atypical NAD) have a pediatric onset, while a third form of dystonia–parkinsonism has an adult onset [122]. INAD (NBIA2A, OMIM#256600) begins between 6 months and 3 years of age, presenting with gait disturbance, truncal hypotonia, hyperreflexia, developmental delay, and visual impairment [148]. Atypical NAD (NBIA2B, NBIA/DYT/PARK-*PLA2G6*, OMIM#610217) shows a more variable presentation, generally beginning in early childhood but sometimes as late as the end of the second decade. Initial symptoms include gait instability, ataxia, speech delay, or autistic traits, with progression to dystonia, dysarthria, and neuropsychiatric symptoms (i.e., hyperactivity, impulsivity, emotional lability, and poor attention). Strabismus, nystagmus, and optic atrophy are common in both conditions [148].

DYT-*SQSTM1* (OMIM#617145) is a rare neurodegenerative syndrome caused by variants in *SQSTM1*, which disrupts the function of sequestosome-1, a scaffolding protein critical for autophagy regulation. Biallelic loss-of-function variants have been reported in childhood- or adolescence-onset cases with gait abnormalities, ataxia, dysarthria, dystonia, vertical gaze palsy, and cognitive decline [149,150,151]. Less commonly, psychiatric disorders, iridoplegia, and hypergonadotropic hypogonadism have also been observed [152].

Table 4 outlines the key genes identified in the literature as being associated with complex dystonia with ataxia in the pediatric population [6,122,135,136,137,138,139,140,141,142,143,144,145,146,147,148,149,150,151,152].

### 5.3. Complex Dystonias in Combination with Parkinsonism

In pediatric populations, early-onset dystonia and parkinsonism can be caused by a range of underlying genetic and metabolic conditions, including WD, inborn errors of manganese transport, Huntington’s disease (HD), and NBIA, including COASY-associated neurodegeneration (CoPAN) and Kufor Rakeb disease.

WD (DYT-*ATP7B*, OMIM #277900) is a rare autosomal recessive genetic disorder of copper metabolism caused by disease-causing variants in the *ATP7B* gene [121]. Symptoms typically appear in the first or second decade of life, with nearly half of patients initially presenting with neurological features, such as flapping tremor and dysarthria, and psychiatric symptoms [153,154,155]. Dystonia is a common manifestation, affecting approximately two-thirds of patients, and can range from focal forms (i.e., blepharospasm, cervical dystonia, or risus sardonicus) to multifocal or generalized as the disease progresses [153,156]. Other movement disorders, including parkinsonism, ataxia, and chorea, have also been reported [157].

IEMs affecting manganese transport, such as disease-causing variants in the *SLC30A10* and *SLC39A14* genes, are treatable conditions often characterized by movement disorders [156]. Biallelic variants in *SLC30A10* cause a syndrome marked by early-onset dystonia, parkinsonism, hepatic cirrhosis, polycythemia, and hypermanganesemia (hypermanganesemia with dystonia, DYT/PARK-*SLC30A10*, OMIM#613280). Symptoms typically begin in early childhood with gait disturbances (“cock-walk gait”) due to limb dystonia, which often progresses to generalized dystonia, central hypotonia, and impaired motor skills [158,159,160]. In rare cases, spastic paraplegia may occur, while cognitive development is usually preserved [161]. Similarly, biallelic variants in *SLC39A14* result in an early-onset progressive form of dystonia–parkinsonism correlated to hypermanganesemia (OMIM#617013) [157,162]. Affected children often show developmental delay, progressive dystonia, and bulbar dysfunction, typically manifesting in infancy or early childhood [163]. In some cases, parkinsonian features such as hypomimia, tremor, and bradykinesia also emerge [157,162,163].

Beyond IEMs, genetic neurodegenerative diseases can also present with prominent movement disorders, including dystonia and parkinsonism. Among them, HD (OMIM#143100) is an autosomal dominant disorder caused by a CAG trinucleotide repeat expansion in the gene *HTT* [164]. While chorea is the hallmark motor feature in adult-onset HD, juvenile-onset HD is a rare subset of the disease characterized by a predominance of mixed motor symptoms, including dystonia (particularly cervical dystonia) and parkinsonism. In addition to motor symptoms, developmental delay, cognitive dysfunction, learning disabilities, and behavioral or psychiatric manifestations have been reported [112,165].

Finally, certain subtypes of NBIA are also characterized by dystonia and parkinsonism. One example is CoPAN (OMIM#615643), an autosomal recessive form of NBIA caused by mutations in the *COASY* gene. It is characterized by early-onset gait difficulties and learning and cognitive impairment, accompanied by movement disorders, such as spastic-dystonic paraparesis, oromandibular dystonia, parkinsonism, and dysarthria [6,166,167]. Kufor Rakeb disease (OMIM#606693) is an NBIA caused by pathogenic variants in *ATP13A2*. Affected patients manifest juvenile-onset parkinsonism, dystonia, myoclonus, supranuclear gaze palsy, spasticity, and dementia [167,168].

Table 5 outlines the key genes identified in the literature as being associated with complex dystonia with parkinsonism in the pediatric population [6,112,121,153,154,155,156,157,158,159,160,161,162,163,164,165,166,167,168].

### 5.4. Complex Dystonias in Combination with Chorea

Lesch–Nyhan disease (LND, DYT/CHOR-*HPRT*, OMIM#300322) is a rare X-linked recessive disorder caused by a deficiency of the hypoxanthine–guanine phosphoribosyl transferase (HPRT) enzyme, crucial for the purine salvage pathway [169]. This enzymatic deficiency results in elevated levels of uric acid, neurological and behavioral symptoms, including IDD, self-injurious behavior, and severe motor dysfunction (such as chorea, choreoathetosis, athetosis, dystonia, opisthotonus, and ballism) due to basal ganglia abnormalities [122,170]. Most patients with LND exhibit extrapyramidal signs and generalized dystonia [170,171].

Additionally, IEMs such as organic acidurias (OADs), are a frequent cause of dystonia and chorea in children, combined with encephalopathy. These inherited neurometabolic disorders arise from enzyme deficiencies in amino acid degradation, resulting in the toxic accumulation of organic acids in the brain and other tissues [172,173]. They commonly present in infancy, but late-onset forms can occur during childhood or adulthood. OADs are categorized into classical forms, which typically manifest early with acute encephalopathic crises triggered by infections or immunizations, and cerebral forms, which present later with progressive neurological symptoms, including developmental delay, IDD, seizures, dystonia, choreoathetosis, spasticity, ataxia, and macrocephaly [6,123,172]. OADs include various conditions, such as isolated methylmalonic acidemia (i.e., DYT/CHOR-*MMUT*—OMIM#251000, *MMAA*—OMIM#251100, *MMAB*—OMIM#251110, *MMADHC*—OMIM#277410 and OMIM#620953, and *MCEE*—OMIM#251120), propionic acidemia (i.e., DYT/CHOR-*PCCA* and DYT/CHOR-*PCCB*—OMIM#606054), glutaric acidemia type 1 (DYT/CHOR-*GCDH*—OMIM#23170), and ethylmalonic encephalopathy (*ETHE1*—OMIM#602473) [122,123,172].

Table 6 outlines the key genes identified in the literature as being associated with complex dystonia with chorea in the pediatric population [6,122,123,169,170,171,172,173].

### 5.5. Complex Dystonias in Combination with Epilepsy

Recent studies highlight that pediatric movement disorders, like dystonia, share a genetic basis with certain epilepsy syndromes [174]. These conditions are highly heterogeneous and, while more monogenic defects are being identified, research into their clinical and genetic characteristics in dystonia cohorts remains limited [175].

Developmental and epileptic encephalopathies (DEEs) are a group of disorders that often involve both seizures and movement abnormalities. These conditions are characterized by drug-resistant seizures and neurodevelopmental delay, which typically begin in the neonatal period or infancy [174,176]. Dystonia may be a notable feature in many genetically determined DEEs and can manifest as focal, segmental, or generalized forms, occasionally escalating into life-threatening episodes such as SD or dystonic storms [174]. The genes implicated in DEEs with dystonia include *SCN1A* and *SCN8A* (sodium channel genes—OMIM#619317 and #614558, respectively), *GABRA1* (gamma-aminobutyric acid-related gene—OMIM#615744), *GRIN1* (glutamatergic-related gene—OMIM#138249), *TBC1D24* and *DNM1* (synaptic vesicle dynamics—OMIM#613577 and #602377, respectively), and others with diverse functions, such as *UBA5* (protein posttranslational modification—OMIM#610552), *SPTAN1* (cytoskeleton organization—OMIM#182810), *WWOX* (apoptosis and tumor suppression—OMIM#605131), *GNAO1* (synaptic transmission modulation), and *FOXG1* (OMIM#164874) [174,175,177,178,179,180].

Table 7 outlines the key genes identified in the literature as being associated with complex dystonia with epilepsy in the pediatric population [174,175,176,178,179,180].

### 5.6. Complex Dystonias in Combination with Hearing Impairment

Certain genetic conditions, often associated with mitochondrial dysfunction or specific gene disease-causing variants, can cause both auditory disturbances and dystonia. Notable examples include deafness–dystonia syndromes (DDSs), which encompass conditions such as Mohr–Tranebjaerg syndrome, Woodhouse–Sakati syndrome, *ACTB*-related disorders, and methylmalonic aciduria, alongside pathogenic variants in the *BCAP31* gene.

DDSs are rare conditions characterized by the combination of sensory–neural hearing loss and dystonia, often linked to mitochondrial dysfunction due to variants in nuclear or mitochondrial DNA [181]. Several genetic disorders have been identified as causes of DDS, each presenting with distinct but overlapping clinical features. Mohr–Tranebjaerg syndrome (DYT-*TIMM8A*, OMIM#304700), an X-linked disease caused by disease-causing variants in *TIMM8A*, is a key example where deafness and dystonia are hallmark features accompanied by variable symptoms such as optic atrophy, pyramidal signs, psychiatric disturbances, and cognitive decline [181,182]. Similarly, Woodhouse–Sakati syndrome (caused by biallelic variants in the *C2orf37* gene, also known as NBIA/DYT-*DCAF17*, OMIM#241080) presents with multisystem involvement, including hypogonadism, diabetes, and alopecia along with neurological symptoms such as deafness, dystonia, and IDD [181,183]. Additionally, both *SUCLA2*-related mitochondrial DNA depletion syndrome (DYT-*SUCLA2*—OMIM#612073) and *SERAC1*-related MEGDEL (3-methylglutaconic aciduria with deafness–encephalopathy–Leigh-like—OMIM#614739) syndrome are autosomal recessive disorders: methylmalonic aciduria (due to *SUCLA2* variants or other metabolic defects) typically manifests with neonatal encephalopathy, severe hypotonia, developmental delay, dystonia, and early-onset sensorineural hearing loss, and Leigh-like syndrome (caused by variants in the *SERAC1*) presents with progressive neurological decline, deafness, dystonia, and early developmental delay [181]. Moreover, DDSs can also arise from heterozygous variants in *ACTB* (OMIM#607371), which are associated with congenital deafness, early-onset focal dystonia that rapidly progresses to a multifocal pattern, and IDD or developmental delay [22,184].

Pathogenetic variants in the *BCAP31* gene also lead to a severe congenital neurological phenotype, characterized by deafness, dystonia, and central hypomyelination (OMIM#300475). Other reported features are strabismus, optic atrophy, seizures, and microcephaly [185,186,187].

Table 8 outlines the key genes identified in the literature as being associated with complex dystonia with hearing impairment in the pediatric population [22,181,182,183,184,185,186,187].

### 5.7. Complex Dystonias in Combination with Ocular Impairment

Several conditions can cause both visual disturbances (such as optic neuropathy/atrophy) and dystonia in children. In particular, mitochondrial inherited disorders (MIDs), a heterogeneous group of genetic disorders caused by disease-causing variants in either mitochondrial or nuclear DNA, are commonly associated with multisystem involvement, including neurological manifestations and ophthalmic symptoms such as optic neuropathy and progressive external ophthalmoplegia [188,189]. Movement disorders, such as dystonia, ataxia, myoclonus, and parkinsonism, are common and may occur alone or alongside other symptoms. Dystonia is the most reported movement disorder in several MIDs, such as *POLG*-related disorders and mitochondrial encephalopathy, lactic acidosis, and stroke-like episodes (MELAS) [188,190]. Specifically, Leigh syndrome, caused by pathogenic variants in the mitochondrial DNA or various nuclear genes, manifests with developmental delay, hypotonia, dystonia, ataxia, and optical atrophy [188,191,192]. In some cases, a treatable defect in the cerebral thiamine transporter (*SLC19A3*—OMIM#606152) leads to a Leigh-like presentation, with subacute to acute encephalopathy, dystonia, dysarthria, dysphagia, regression of developmental milestones, external ophthalmoplegia, ataxia, and seizures. As the disease progresses, it may result in parkinsonism and quadriplegia [122,137,157].

Additionally, infantile- or childhood-onset dystonia combined with optic atrophy (OMIM#617282) has been linked to disorders of mitochondrial lipid metabolism caused by biallelic variants in the *MECR* gene [193,194].

Table 9 outlines the key genes identified in the literature as being associated with complex dystonia with ocular impairment in the pediatric population [122,137,188,189,190,191,192,193,194].

## 6. Paroxysmal Dyskinesia

Paroxysmal movement disorders (PxMD) are a heterogeneous group of disorders characterized by the occurrence of sudden, discrete, involuntary, episodic hyperkinetic movements (including dystonia, dyskinesia, chorea, and/or ataxia), often triggered by specific stimuli and followed by a return to neurological baseline [22,109]. They were traditionally classified based on interictal neurological findings, distinguishing “primary” (now recognized as genetic) from “secondary” forms (caused by structural, metabolic, or other conditions), where additional neurological signs were expected. More recent evidence has questioned this distinction, favoring a classification based on the presence or absence of additional neurological features [62]. The onset of paroxysmal dyskinesias is usually in childhood, and they very rarely arise after the age of 18 years. In such cases, they are likely due to an underlying organic lesion of the central nervous system [195]. According to the triggering factors, paroxysmal dyskinesia can be subdivided into three main groups: PKD, PED, and paroxysmal nonkinesigenic dyskinesia (PNKD) [196]. However, PxMD are characterized by a wide heterogeneity of clinical features, and the phenotype may frequently encompass different neurological entities, such as epilepsy and epileptic encephalopathy, neurodevelopmental disorders, and hemiplegic migraine [109].

PKD episodes are triggered by sudden movement, involving brief (<1 min), self-limiting, and very frequent (up to hundreds of times per day) episodes of dystonic/choreiform posturing [197]. *PRRT2*-related dyskinesia (PxMD-*PRRT2*—OMIM#602066) is the most common type of paroxysmal movement disorder, more prevalent in males, and it is caused by heterozygous variants in the *PRRT2* gene [112,198,199]. The gene has been identified within the 16p11.2 region and encodes proline-rich transmembrane protein 2, a pre-synaptic protein widely expressed in the cerebellum, basal nuclei, and neocortex [198,200]. The onset of the paroxysmal disorder is typically preceded by epilepsy, which is usually self-remitting within 2 years of age [62]. Episodes are very brief, usually lasting less than 1 min, and are often preceded by a sensory aura at the initial site of the attacks [201]. In half of the cases, the attacks have a clear kinesigenic trigger (i.e., sudden movements, intention to move, and/or acceleration) and involve the face, trunk, and limbs, often starting unilaterally and tending to generalize. The frequency can vary from hundreds per day to just one or two per year. However, the episodes generally become less frequent with age and may even resolve completely in adulthood, regardless of treatment [201,202]. Biallelic *PRRT2* variants have been associated with a more severe phenotype, encompassing IDD, persistent PKD attacks, autism spectrum disorder, ADHD, epilepsy, migraine, PNKD, or episodic ataxia [62]. *PRRT2*-negative cases of PKD may be partially ascribable to monoallelic pathogenic variants in the *TMEM151A* gene (OMIM#620108) [203]. The protein function is still unknown, although it has been proposed that it might be either an endoplasmic reticulum-associated calcium channel or a regulator of calcium sensors in the SNARE complex [204]. The phenotype differs from PxMD-*PRRT2* in terms of attack duration (typically lasting 10–30 s), onset, which usually occurs during childhood or early adolescence, and the more common facial involvement [62]. Recently, paroxysmal kinesigenic movements have also been reported in patients with heterozygous variants in genes such as *RHOBTB2* (OMIM#607352) and *SCN8A*, which are typically related to epilepsy and DEEs [205,206,207].

PED attacks are hyperkinetic movements that last longer (5–30 min, rarely up to 2 h), usually occur several times per week, and are triggered or exacerbated by sustained exercise, but also by fasting, stress, sleep deprivation, cold, muscle vibration, or passive movements [195,196,202]. These disorders are frequently related to heterozygous variants of *SLC2A1* (PxMD-*SLC2A1*, dystonia 9, DYT9, OMIM#601042) [208,209]. The gene is located on chromosome 1 (1p34.2) and encodes the major glucose transporter in the brain [210]. Alterations in this protein lead to a reduction in available glucose in the brain [211]. In some patients, the disorder may progress to spastic paraplegia [212]. Several other neurological presentations may characterize the phenotype, such as seizures, DEE, migraine, ataxia, and hereditary spastic paraplegia [109,212,213]. Dietary strategies to bypass the GLUT1 transporter defect include the ketogenic diet and triheptanoin [214]. PED has also been reported in a few cases involving biallelic variants of *TBC1D24*, a gene that is usually linked to DEE, non-syndromic hearing loss, myoclonus, and DOORS (deafness, onychodystrophy, osteodystrophy, impaired intellectual development, and seizure syndrome) syndrome. The disorder is characterized by childhood-onset of paroxysmal dyskinetic episodes associated with epilepsy, myoclonus, ataxia, dysmetria, and dysarthria [215,216]. In addition, paroxysmal hyperkinetic movements have been described in patients with biallelic variants of *ECHS1* (OMIM#602292), leading to a Leigh-like syndrome and a PED phenotype [217,218].

Finally, paroxysmal dyskinetic episodes have been reported without movement- or exertion-related trigger. In these cases, the trigger may be a specific condition (i.e., exertion, fatigue, ill health/fever, menstruation, and psychological stress) or the ingestion of methylglyoxal-containing foods (i.e., alcohol, coffee, tea, and chocolate) [22]. The episodes last from minutes to few hours, occur only a few times per year, and progress from early dystonic phenomena to choreiform movements and abnormal speech due to facial involvement [212]. This form (PxMD-*PNKD*—OMIM#118800) is frequently related to *PNKD*, a gene involved in the cellular stress response pathway and redox homeostasis [219]. The *PKND*-related presentation is not associated with epilepsy or neurodevelopmental disorder, in contrast to the PNKD phenotype caused by *KCNMA1* (OMIM#609446) [109]. These latter forms are caused by heterozygous variants in the *KCNMA1* gene, which is frequently implicated in various types of seizures that can coexist within the same phenotype [220,221].

In the last decade, numerous genetic disorders have been reported to encompass in their phenotype recurrent episodes of dystonia and/or chorea (i.e., *ADYC5*, *GCH1*, and *ATP1A3*). In these cases, the episodes of PxMD are usually associated with other features and do not represent the predominant clinical manifestation [62]. Given that their main phenotype has a different presentation, they have been addressed in the previous section.

Table 10 contains a summary of the main genes involved in paroxysmal dyskinesias in the pediatric population, including the clinical features, neuroimaging findings, and treatment options [22,62,109,112,195,196,197,198,199,200,201,202,203,204,205,206,207,208,209,210,211,212,213,214,215,216,217,218,219,220,221].

## 7. Status Dystonicus

SD, also known as dystonic storm, is a life-threatening condition characterized by an acute worsening of generalized dystonia, often accompanied by other hyperkinetic movements [5,222]. SD represents the most severe form of dystonia and is considered a neurological emergency in movement disorders, with a mortality rate of 10–12.5% [223]. SD involves severe and prolonged dystonic posturing, leading to significant morbidity, including metabolic complications. The muscle breakdown (rhabdomyolysis) can subsequently result in renal failure. Additional complications include hypertension, tachycardia, tachypnea, autonomic instability, and bulbar dysfunction, which may lead to respiratory failure, further increasing its high mortality rate [5,224].

Although a significant number of cases have been reported in adults, approximately 60–80% of SD cases occur in children and adolescents, particularly those with pre-existing dystonia associated with neurodevelopmental syndromes [223,224,225]. These underlying conditions range from acquired causes, such as dyskinetic cerebral palsy, to monogenic disorders, including isolated genetic dystonia (i.e., DYT-*TOR1A*), combined dystonia (i.e., DYT/PARK-*TH* and PKDYS1), and complex dystonia (including inherited metabolic disorders such as glutaric aciduria type 1, LND, NBIA/DYT-*PANK2*) [5,224]. In some cases, SD may be the first manifestation of a movement disorder in other conditions, including infectious or immune-mediated central nervous system disorders (i.e., encephalitis), neurotransmitter disorders, and post-traumatic causes [224,226].

SD is often triggered by fever, infection, medication changes, or other causes like trauma, anesthesia, surgery, metabolic abnormalities, dehydration, stress, and hormonal fluctuations [5,224,225,227].

Management follows a stepwise approach. In the first 24 h, treatment focuses on supportive care, identifying and managing potential triggers, and first-line therapy with intravenous midazolam. If needed, propofol anesthesia is used, followed by barbiturates and non-depolarizing neuromuscular blockers like pancuronium [5,227]. Early DBS of the GPi or the posterior–ventrolateral region can be considered, or if unavailable, intrathecal baclofen or surgical options like pallidotomy [5,222,223]. Over the next 2–4 weeks, treatment aims at long-term control of the dystonia symptoms with anticholinergics, dopamine receptor blockers, tetrabenazine, clonidine, and other medications [5,227].

Beyond the classic presentation, some patients with *GNAO1* variants may exhibit a dystonic–dyskinetic status, characterized by the exacerbation of fluctuating and often combined movement disorders, including chorea, dystonia, athetosis, ballism, dyskinesia, and myoclonus [114,228]. This severe neurological condition can lead to progressive motor regression and loss of previously acquired skills. It is also frequently associated with significant medical complications, such as hyperthermia, rhabdomyolysis, elevated creatine kinase levels, joint dislocations, fractures, and sepsis. Notably, in these patients, DBS has shown effectiveness in controlling and preventing these severe exacerbations [228,229].

## 8. Treatment

The first step in treating pediatric genetic dystonias is to identify the underlying etiology to determine whether pathogenesis-targeted treatments are available. This is the case of WD, for which treatment includes lifelong administration of copper-chelating agents (such as penicillamine and trientine) and inhibitors of intestinal copper absorption (zinc salts) [230]. Similarly, chelation therapy with intravenous disodium calcium edetate infusion leads to a dramatic clinical improvement and decrease in manganese levels in *SLC30A10*- and *SLC39A14*-related disorders. Iron supplementation is also helpful, as it competes for the same transporter as manganese (see Table 5) [231].

Chelation therapy is recognized as an effective treatment for NBIA, with the primary iron chelators being deferiprone, deferasirox, and desferrioxamine (also known as deferoxamine). Among these, deferiprone is regarded as the most efficacious due to its low molecular weight, favorable octanol–water partition coefficient, and lipophilic characteristics, which facilitate its ability to cross the blood–brain barrier and specifically target cerebral iron deposits [232]. Indeed, in a large multicentric, randomized, double-blind, placebo-controlled trial conducted on 88 patients with PKAN, followed by an open-label extension, Klopstock and colleagues evaluated the efficacy of deferiprone (30 mg/kg per day) and proved a slower progression of the disease, evaluated with the Barry–Albright Dystonia scale [233] (See Table 3).

Another targeted therapy is Miglustat (OGT 918, N-butyl-deoxynojirimycin), an iminosugar that inhibits glucosylceramide synthase, an enzyme involved in the early stages of glycosphingolipid synthesis. A key advantage of Miglustat is its ability to cross the blood–brain barrier, which makes it particularly suitable for treating neurological manifestations. Originally approved by the U.S. Food and Drug Administration (FDA) for the treatment of Gaucher disease, it was later indicated for the management of progressive neurological symptoms in both adults and children with NPC (see Table 4) [234].

Very recently, gene therapy has been approved by the European Medicines Agency (EMA) and the FDA for patients with AADC (aromatic L-amino acid decarboxylase) deficiency with a genetically confirmed diagnosis [235,236]. It consists of stereotactic delivery of adenoviral vectors containing human DDC copies, administered in the putamen [237,238] or in the substantia nigra and ventral–tegmental areas [239]. The treatment resulted in dramatic and persistent motor improvements (especially in younger patients and lasting more than 5 years) [237], improved cognitive performances in patients with moderate phenotypes [238], and an increased dopamine production confirmed by cerebral spinal fluid markers and positron emission tomography scans/tractography in all patients (see Table 2) [237,238,239].

However, since in most cases the exact mechanisms of dystonia remain poorly understood, treatment is primarily symptomatic, aiming to relieve abnormal movements and postures, associated pain and discomfort, contractures, and orthopedic complications [240]. Treatment often requires a multidisciplinary approach, including physical and speech therapy, oral medications, chemodenervation with botulinum toxin (BT) injections, and, in refractory forms, neurosurgical interventions, such as DBS. In children, dystonia is more commonly generalized/multifocal, and oral medications are primarily used for these forms, sometimes combined with BT in focal or segmental cases. Management depends on the type and severity of the condition and may involve pharmacological options, such as dopaminergic agents, anticholinergics, benzodiazepines, and muscle relaxants, to alleviate motor symptoms [22,241,242].

BT, produced by *Clostridium botulinum*, binds to specific sites on the presynaptic cholinergic nerve terminal, inhibiting acetylcholine release and causing neuromuscular blockade. BT has seven distinct serotypes (A to G), but only two are approved by the FDA and EMA. Each serotype consists of a 100 kDa heavy chain and a 50 kDa light chain. The heavy chain binds to peripheral cholinergic nerve terminals, facilitating the endocytosis of BT [243]. Once inside the cytoplasm, the light chain is released and inhibits SNARE proteins, preventing the release of acetylcholine. For BT therapy, selecting the appropriate muscles for injection and administering the neurotoxin with precision are crucial for a successful treatment outcome. The effects of a BT injection typically begin within 1 week and last for about 2.5–3 months [241,243]. BT is commonly used for focal and segmental dystonia. However, its use in the pediatric population is limited due to the rarity of these forms and the lack of randomized controlled trials [22,241].

Among dopaminergic drugs, levodopa is the first-line treatment for dopa-responsive dystonia, such as *GCH1*-, *TH*-, and *SPR*-related disorders, and may also provide some benefit in combined dystonia–parkinsonism [22]. It is generally used at a low dose with good response and is well-tolerated, although patients may develop nausea, drowsiness, and lightheadedness. Recent findings suggest that long-term carbidopa–levodopa therapy can moderately improve motor function. Some patients can develop levodopa-induced dyskinesia, but it usually presents at the initiation of treatment and is the result of unusually high doses (see Table 2) [59,244]. However, apart from dopa-responsive dystonia, levodopa is generally ineffective for other types of dystonia, likely due to differences in their underlying pathophysiological mechanisms, which are not primarily linked to dopamine biosynthesis [22].

Anticholinergic drugs, such as trihexyphenidyl, are effective in treating generalized and multifocal dystonia. However, their use is often limited by side effects like dry mouth, blurred vision, and cognitive impairment [240]. These medications are most beneficial for patients with isolated generalized dystonia rather than for those with combined forms of dystonia. Starting with a low dose and gradually increasing it over several weeks can help minimize the side effects and improve tolerability [11,22].

Among the GABAergic agonists, baclofen may be used in patients with oromandibular dystonia, as well as in those with segmental or generalized dystonia, spasticity, and dystonic-choreoathetoid cerebral palsy. More data support the use of intrathecal and intraventricular baclofen infusions [11,22,245]. Benzodiazepines, particularly clonazepam, are used for muscle relaxation and myoclonus–dystonia but are limited by sedation, tolerance, and dependency risks. Other drugs, such as zonisamide, zolpidem, and sodium oxybate, have shown efficacy in myoclonus–dystonia but are not considered standard treatments [11,22].

Severe cases of drug-resistant generalized, segmental, and some focal dystonias may be candidates for DBS. The primary stimulation target is the GPi, although efficacy has also been observed with stimulation of the subthalamic nucleus and thalamus [246]. Notably, GPi-DBS has proven to be a safe and effective treatment for isolated genetic dystonias, including those associated with *TOR1A*, *KMT2B*, and *THAP1* variants. Additionally, combined dystonias may also respond well to GPi-DBS (see Table 1, Table 2 and Table 3) [22,247].

Potential predictors of a good response to DBS include younger age at dystonia onset, disease severity, and shorter disease duration before treatment, with earlier intervention often leading to better outcomes. Conversely, severe speech impairment and older age have been linked to less favorable DBS outcomes [22,247].

Among specific treatments, paroxysmal dyskinesias require distinct therapeutic approaches involving antiseizure medications (ASMs) [248,249]. In particular, PKD is typically treated with low-dose carbamazepine, which is highly effective, while other beneficial ASMs include other sodium channel blockers such as oxcarbazepine, phenytoin, and lacosamide [250]. Conversely, in PNKD, ASMs are generally less effective, and treatment consists of low-dose benzodiazepines. For PED, dietary modifications, particularly a ketogenic diet with L-carnitine supplementation, constitute the mainstay of treatment (see Table 10) [22].

## 9. Limitations

One of the limitations of this review concerns the classification of genes with broad and heterogeneous clinical presentations. For many of the genes discussed, published reports describe multiple phenotypes, which led us to focus on the most frequently reported or predominant clinical presentation in each case. To avoid redundancy, we did not reiterate all possible phenotypes in every section. This issue is particularly relevant to the section on complex dystonias, where we adopted a didactic approach based on the predominant phenotype. However, it is important to acknowledge that many of these disorders are characterized by overlapping movement disorder features.

Furthermore, the pathogenicity of numerous variants remains poorly defined in the literature, due in part to a lack of functional studies that could offer deeper insights into the pathophysiological mechanisms underlying the observed phenotypes. In this regard, integrating functional analyses of variant-specific effects will be essential to improve genotype–phenotype correlations.

## 10. Conclusions

Genetic dystonias in pediatric patients encompass a broad spectrum of disorders, each with distinct clinical and molecular characteristics. The correlation between specific genes and clinical presentations enables a more targeted diagnostic approach, allowing clinicians to suspect the underlying genetic cause based on symptomatology. Identifying the precise genetic etiology is crucial, as it offers valuable insights into disease prognosis and potential treatment strategies.

Advancements in genetic testing have significantly improved our ability to diagnose dystonias at an early stage. However, challenges persist in fully understanding genotype–phenotype correlations. Certain genetic forms of dystonia demonstrate distinct therapeutic responses, highlighting the need to refine our understanding of how different variants influence the disease course and treatment outcomes.

Future research should focus on further characterizing the relationship between genotype and phenotype, with the goal of developing personalized treatment strategies tailored to both the implicated gene and the specific variant involved. A deeper understanding of these mechanisms will not only enhance our ability to predict disease progression but also pave the way for more effective, gene-targeted therapies, ultimately improving the quality of life for affected children.

## Figures and Tables

**Table 1:** Genes associated with isolated dystonia: clinical features, neuroimaging, and treatment options.

Gene	Inheritance	Age at Onset	Dystonia Onset	Course of Dystonia	Dystonia Progression	Cognitive Development	Neuropsychiatric Features	Brain MRI	Treatment Options
*TOR1A*	AD	Childhood	Focal	Progressive	Onset in lower limbs and generalizing with sparing of larynx and neck	Normal	Gait difficulties	Nonspecific findings	Symptomatic GPi DBS
*THAP1*	AD	Childhood to adulthood	Segmental	Progressive	Onset in cranio-cervical region/upper limbs and generalizing with larynx involvement	NR	Speech impairment, dysarthria, dysphonia	GP hypointensities	Symptomatic GPi DBS
*KMT2B*	AD	Childhood	Focal	Progressive	Onset in lower limbs with caudocranial generalization	DD/IDD	Microcephaly, seizures, and mixed MD with choreoathetosis or myoclonus	GP hypointensities	Symptomatic GPi DBS
*HPCA*	AR	Childhood	Focal	Slowly progressive	Onset distally and generalizing with prominent cranio-cervical involvement	Normal or DD/IDD	Mood disorder, seizures, mixed MD with choreoathetosis	Normal	Symptomatic
*ANO3*	AD	Childhood to adulthood	Multifocal/ segmental	Progressive	Cranio-cervical onset with generalizing to upper limbs	Normal, occasionally ID	Anxiety, depression, sleep disturbance, seizures, mixed MD with chorea, myoclonus	Normal	Symptomatic GPi-DBS
*TUBB4A*	AD	Early childhood to adulthood	Focal	Progressive	Laryngeal onset with generalizing to neck, face, and limbs	DD	Dysarthria, whispered dysphonia, mixed MD with choreoathetosis, ataxia, spasticity	H-ABC	Symptomatic
*GNAL*	AD/AR	Childhood to adulthood	Focal/ Segmental; rarely generalized	Progressive	Onset in laryngeal and cranio-cervical regions, occasionally generalizing	Normal, occasionally IDD	Frequent mixed MD with tremor or parkinsonism; in biallelic variants, dysmorphic features, sensorineural hearing	Normal	Symptomatic GPi-DBS
*VPS16*	AD/AR	Childhood	Generalized	Slowly progressive	Onset in oromandibular, bulbar, cervical, upper limb regions, and generalization	Normal or DD/IDD	Anxiety, depression, and emotional lability, neurodevelopmental disorders	Normal or BG hypointensities (iron deposition)	Symptomatic GPi-DBS
*VPS41*	AR	Infancy	Generalized	Progressive	NR	DD/IDD	Motor dysfunction with ataxia, nystagmus, speech delay, optic atrophy, and axonal neuropathy	Normal or cerebellar vermis atrophy	NR
*EIF2AK2*	AD/AR	Childhood	Generalized	Progressive	Onset either in the upper or lower limbs or trunk, and generalizing	Normal or DD/IDD	MD, spasticity, seizures, speech impairment, and neurological regression in the context of febrile illness	Normal or hypomyelination/delayed myelination, thin CC, lower medullary lesions	Symptomatic

Legend: AD = autosomal dominant; *ANO3* = Anoctamin 3; AR = autosomal recessive; BG = basal ganglia; CC = corpus callosum; DBS = Deep brain stimulation; DD = developmental delay; DTI = Diffusion tensor imaging; *EIF2AK2* = eukaryotic translation initiation factor 2-alpha kinase 2; GNAL = G protein subunit alpha L; GP = globus pallidum; GPi = internal globus pallidum; H-ABC = hypomyelination with atrophy of the basal ganglia and cerebellum; *HPCA* = hippocalcin; IDD = intellectual developmental disorder; *KMT2B* = lysine methyltransferase 2B; MD = movement disorder; MRI = magnetic resonance imaging; NR = not reported; *THAP1* = Thanatos-associated protein domain-containing apoptosis-associated protein 1; *TOR1A* = torsin family 1 member A; *TUBB4A* = tubulin beta 4A class Iva; *VPS16* = VPS16 core subunit of CORVET and HOPS complexes; *VPS41* = VPS41 subunit of HOPS complex.

**Table 2:** Genes associated with combined dystonia: clinical features, neuroimaging, and treatment options.

Gene	Inheritance	Age at Onset	Dystonia Onset	Course of Dystonia	Dystonia Progression	Other MDs	Cognitive Development	Neuropsychiatric Features	Brain MRI	Treatment Option
*GCH1*	AD	Infancy (atypical form) to childhood	Focal limb dystonia	Progressive	Rostrally spreading until generalized	Parkinsonism	Normal	Anxiety, depression	Normal	Levodopa
	AR	Neonatal to early childhood	Dystonic dysarthria, focal limb or cervical dystonia	Progressive	Neurological deterioration and generalized dystonia	Parkinsonism	Normal to severe IDD	NR	Normal	Levodopa, Phe-restricted diet
*TH*	AR	Neonatal period/infancy to childhood	Focal lower limb dystonia	Progressive	From one leg to the other leg, arms, trunk, face, and oropharyngeal musculature	Parkinsonism, tremors, myoclonus, OGC	DD/IDD	Behavioral disturbances, encephalopathy	Normal	Levodopa
*SPR*	AR	Infancy	Focal and generalized	Progressive	NR	Parkinsonism, tremors, dysarthria, distal hypertonia, OGC	DD/IDD	Psychiatric disorders, sleep disturbances	Normal or nonspecific findings	Levodopa
*PRKRA*	AR	Childhood to adolescence	Focal limb or cervical dystonia	Progressive	Focal limb or cervical dystonia spreading until generalized	Parkinsonism, tremors	Normal, DD/IDD	Language delay, behavioral disturbances	Normal	Mild to non-response to levodopa
*DDC*	AR	Infancy	Focal limb or cervical dystonia, OGC	Progressive	Generalized	Parkinsonism, OGC, dysarthria, ptosis, choreoathetosis, myoclonic startles	DD/IDD	Anxiety, depression, OCD, ODD, ADHD	Normal	Intraputaminal infusions of eladocagene exuparvovec
*ATP1A3*	AD	Adolescence to early adulthood	Asymmetrical focal dystonia	Slowly progressive with abrupt exacerbations	Rostro-caudal spreading	Parkinsonism, dysarthria, dysphagia, ocular apraxia	IDD	Mood disorders, psychosis, schizoid personality disorder	Normal	Symptomatic
*SLC6A3*	AR	Typical: infancy (6 months) Atypical: childhood to adulthood	Generalized	Progressive	Hyperkinetic disorder that progresses to dystonia–parkinsonism	Parkinsonism, chorea, ballism, orolingual dyskinesia, tremors	Normal	ASD, ADHD, bipolar disorder	Normal	Symptomatic
*VAC14*	AR	Infancy to childhood	Generalized	Progressive	Progression in severity	Parkinsonism	DD/IDD	NR	Normal or T2 hyperintensities of the putamen and caudate nucleus and T2 hypointensity of the pallidum and substantia nigra	Symptomatic or GPi-DBS
*WARS2*	AR	Childhood	Focal limb dystonia	Progressive	Generalized	Parkinsonism, tremors, ballism, myoclonus	Normal to IDD	Aggressive behavior, anxiety, depression, psychosis, epilepsy	Normal	Levodopa
*SGCE*	AD	Childhood	Focal limb or cervical dystonia	Progressive	Increased action dystonia (writer’s cramp)	Myoclonus	Normal to mild IDD	Panic disorder, depression, anxiety disorder, OCD, ADHD	Normal	Symptomatic
*KCTD17*	AD	Childhood to adolescence	Focal upper limb dystonia	Progressive	Spreading to cranio-cervical regions, limbs, trunk, and oromandibular and laryngeal muscles	Myoclonus, distal choreic movements	Normal to mild DD/IDD	Anxiety and social phobia, obsessive traits, depression	Normal	GPi-DBS
*KCNN2*	AD	Childhood	Focal upper limb dystonia (writer’s cramp)	Static	NR	Myoclonus, tremor, ataxia, chorea, tics, nystagmus	Normal to mild IDD	ASD, anxiety, epilepsy, depression, ADHD	Normal to periventricular hyperintensities	Symptomatic
*GRIN2A*	AD	Infancy to childhood	Generalized	Progressive	Progression in severity	Myoclonus, chorea, tremor, ataxia	DD/IDD	Speech and language disorder, epilepsy, ADHD, ASD	Normal or unspecific findings	Symptomatic
*YY1*	AD	Childhood	Focal upper limb dystonia, *laterocollis*	Static	NR	Myoclonus	Normal	ADHD	Normal	Symptomatic
*ADCY5*	AD/AR	Infancy to childhood	Generalized	Static to mild improvement	Progression in severity	Myoclonus, chorea	Normal to mild IDD	OCD, depression, anxiety, and phobias	Normal or nonspecific findings	Caffeine, acetazolamide, clonazepam, methylphenidate, and DBS
*GNAO1*	AD	Infancy to early childhood	Generalized	Progressive	Progression in severity, frequent status dystonicus	Chorea, ballism, myoclonus	DD/IDD	Epilepsy	Diffuse cortical atrophy, cerebellar atrophy, thinning of CC, focal abnormalities of the BG	Symptomatic, DBS
*NKX2-1*	AD	Infancy	Focal limb dystonia	Static	Improvement during adolescence	Chorea, ataxia	Normal to IDD	Hypotonia, ASD, ADHD, anxiety	Normal	Symptomatic

Legend: AD = autosomal dominant; *ADCY5* = adenylate cyclase 5; ADHD = attention deficit hyperactivity disorder; AR = autosomal recessive; ASD = autism spectrum disorder; *ATP1A3* = ATPase Na^+^/K^+^ transporting subunit alpha 3; BG = basal ganglia; CC = corpus callosum; *DDC* = dopa decarboxylase; DD = developmental delay; *GCH1* = GTP cyclohydrolase 1; *GNAO1* = G protein subunit alpha O1; GPi-DBS = deep brain stimulation of the globus pallidus internus; *GRIN2A* = glutamate ionotropic receptor NMDA type subunit 2A; IDD = intellectual developmental disorder; *KCNN2* = potassium calcium-activated channel subfamily N member 2; *KCTD17* = potassium channel tetramerization domain containing 17; MD = movement disorder; MRI = magnetic resonance imaging; *NKX2-1* = NK2 homeobox 1; NR = not reported; OCD = obsessive compulsive disorder; ODD = oppositional defiant disorder; OGC = oculogyric crises; Phe = phenylalanine; *PRKRA* = protein activator of the interferon-induced protein kinase; *SGCE* = epsilon-sarcoglycan; *SLC6A3* = solute carrier family 6 member 3; *SPR* = sepiapterin reductase; *TH* = tyrosine hydroxylase; *YY1* = YY1 transcription factor; *WARS2* = tryptophanyl tRNA synthetase 2, mitochondrial; *VAC14* = VAC14 component of PIKFYVE complex.

**Table 3:** Genes associated with complex dystonia with spasticity: clinical features, neuroimaging, and treatment options.

Gene	Inheritance	Age at Onset	Dystonia Onset and/or Distribution	Other MD	Course of Dystonia	Other Systemic Features/Associated Diseases	Cognitive Development	Neuropsychiatric Features	Brain MRI	Treatment Option
*ADAR*, *TREX1*	AR/AD	Infancy	Generalized	Spasticity, ataxia, spastic paraparesis	Slowly progressive	AGS, chilblain, sterile pyrexias, hepatosplenomegaly, cortical blindness	IDD	Irritability, microcephaly, seizures, encephalopathy	Calcification of the putamen, GP, and thalamus	Symptomatic
*RNASEH2A*, *RNASEH2B*, *RNASEH2C*, *SAMHD1*, *IFIH1*	AR									
*PANK2*	AR	Classic PKAN: infancy	Focal progressing to generalized	Parkinsonism, spasticity, choreoathetosis	Progressive	PKAN, pigmentary retinopathy, acanthocytosis	DD/IDD	Dyspraxia, dysarthria, speech disorder	“*Eye of the tiger*” sign	Symptomatic, DBS, iron chelation
		Atypical PKAN: childhood			Slowly progressive			OCD, dysarthria, Tourette disorder		
*C19orf12*	AR/AD	Childhood to early adulthood	Focal/Multifocal progressing generalized	Spasticity, parkinsonism	Progressive	MPAN, optic atrophy, peripheral neuropathy, bladder and/or bowel incontinence	IDD	Depression, anxiety, OCD, hallucinations, ADHD, dysarthria, dysphagia	Iron deposition in BG and SN	Symptomatic
*FA2H*	AR	Early childhood	Focal progressing to generalized	Ataxia, spasticity	Progressive	FAHN, SPG35, exotropia, optic atrophy	IDD	Seizures, leukodystrophy, mood disorder	Iron deposition in GP pontocerebellar atrophy, thinning of CC	Symptomatic and ablative pallidotomy or thalamotomy

Legend: AD = autosomal dominant; *ADAR* = adenosine deaminase RNA specific; ADHD = attention deficit hyperactivity disorder; AGS = Aicardi–Goutières syndrome; AR = autosomal recessive; BG = basal ganglia; CC = corpus callosum; *C19orf12* = chromosome 19 open reading frame 12; DBS = deep brain stimulation; DD = developmental delay; FAHN = fatty acid hydroxylase-associated neurodegeneration; *FA2H* = fatty acid 2-hydroxylase; GP = globus pallidus; IDD = intellectual developmental disorder; *IFIH1* = interferon induced with helicase C domain 1; MD = movement disorder; MPAN = mitochondrial membrane protein-associated neurodegeneration; MRI = magnetic resonance imaging; OCD = obsessive–compulsive disorder; *PANK2* = pantothenate Kinase 2; PKAN: pantothenate kinase-associated neurodegeneration; *RNASEH2A* = ribonuclease H2 subunit A; *RNASEH2B* = ribonuclease H2 subunit B; *RNASEH2C* = ribonuclease H2 subunit C; *SAMHD1* = SAM and HD domain-containing deoxynucleoside triphosphate triphosphohydrolase 1; SN = substantia nigra; SPG35 = spastic paraplegia-35; *TREX1* = three prime repair exonuclease 1.

**Table 4:** Genes associated with complex dystonia with ataxia: clinical features, neuroimaging, and treatment options.

Gene	Inheritance	Age at Onset	Dystonia Onset and/or Distribution	Other MD	Course of Dystonia	Other Systemic Features/Associated Diseases	Cognitive Development	Neuropsychiatric Features	Brain MRI	Treatment Option
*ATM*	AR	Early childhood to adulthood	Focal/segmental progressing to generalized	Ataxia, myoclonus, chorea, parkinsonism, postural and rest kinetic tremor	Progressive	Ataxia–telangiectasia, immunodeficiency, predisposition to malignancy	Cognitive deterioration	Peripheral neuropathy, oculomotor apraxia, dysarthria	Cerebellar atrophy	Symptomatic
*NPC1*, *NPC2*	AR	Infancy to adulthood	Generalized	Ataxia	Progressive	Niemann–Pick disease, hepatosplenomegaly	DD/IDD	Dysarthria, dysphagia, seizures, gelastic cataplexy, vertical supranuclear gaze palsy	Atrophy of the cerebellar vermis, thinning of the CC, mild cerebral atrophy	Miglustat, symptomatic
*GLB1*	AR	Late childhood to adulthood	Multifocal progressing to generalized	Ataxia, akinetic– rigid parkinsonism, prominent orofacial dystonia	Progressive	GM1 gangliosidosis, skeletal abnormalities, short stature, corneal clouding, facial dysmorphism	IDD	Behavioral/psychiatric disorders	General cerebral atrophy, ventriculomegaly, and/or a “*wish bone*” pattern of iron accumulation	Symptomatic
*FUCA1*	AR	Childhood	Focal progressing to generalized	Dystonic posturing, spasticity	Progressive	Coarse facial features, delayed growth, sinopulmonary infections, visceromegaly, angiokeratoma, dysostosis	Cognitive deterioration	Seizures	Hyperintensity in the BG	Symptomatic
*PLA2G6*	AR	Infancy to early adulthood	Focal progressing to generalized	Parkinsonism, spasticity, nystagmus	Variable	INAD, ANAD, strabismus, optic atrophy	IDD	Speech delay, ASD, ADHD, emotional lability, seizures	Cerebellar atrophy, iron deposition in GP	Symptomatic
*SQSTM1*	AR	Childhood	Facial and lower limb dystonia progressing to generalized	Ataxia, gaze palsy, myoclonus	Progressive	Dysautonomia, delayed growth	Cognitive deterioration	Cerebellar and pyramidal signs, dysarthria, oculomotor involvement	Normal or iron accumulation in the BG	Symptomatic
*DNM1L*	AR/AD	Childhood	Paroxysmal, generalized, or action dystonia	Ataxia, spasticity, nystagmus	Progressive	Hyperlactacidemia optic atrophy	IDD	Epilepsy, dysarthria, microcephaly, sensory and motor axonal neuropathy, neurodegenerative disorder	Nonspecific findings	Symptomatic

Legend: AD = autosomal dominant; ADHD = attention deficit hyperactivity disorder; ANAD = atypical neuroaxonal dystrophy; AR = autosomal recessive; ASD = autism spectrum disorder; *ATM* = ATM serine/threonine kinase; BG = basal ganglia; CC = corpus callosum; DD = developmental delay; *DNM1L* = dynamin 1 like; *FUCA1* = alpha-L-fucosidase 1; *GLB1* = galactosidase beta 1; GP = globus pallidus; IDD = intellectual developmental disorder; INAD = Infantile neuroaxonal dystrophy; MD = movement disorder; MRI = magnetic resonance imaging; *NPC1* = NPC intracellular cholesterol transporter 1; *NPC2* = NPC intracellular cholesterol transporter 2; *PLA2G6* = phospholipase A2 group VI; *SQSTM1* = sequestosome 1.

**Table 5:** Genes associated with complex dystonia with parkinsonism: clinical features, neuroimaging, and treatment options.

Gene	Inheritance	Age at Onset	Dystonia Onset and/or Distribution	Other MD	Course of Dystonia	Other systemic Features/Associated Diseases	Cognitive Development	Neuropsychiatric Features	Brain MRI	Treatment Option
*ATP7B*	AR	Childhood to adulthood	Focal/generalized	Face involvement with *risus sardonicus* “flapping” tremor, parkinsonism, choreoathetosis	Progressive	WD, Liver disease, Kayser-Fleischer corneal rings, low serum copper, and ceruloplasmin	IDD	Depression, bipolar spectrum disorder, personality changes, psychosis	“*Face of the giant panda*” sign	Chelation therapy
*SLC30A10*	AR	Childhood to adolescence	Focal progressing to generalized	“*Cock-walk gait*”, fine tremor, bradykinesia, dysdiadochokinesis	Progressive	Hypermanganesemia, polycythemia, liver disease, darker skin tone	Normal	PICA, dysarthria	Hyperintensity in the BG and cerebellum	Chelation therapy
*SLC39A14*	AR	Infancy to early childhood	Focal progressing to generalized	Spasticity, bulbar dysfunction, parkinsonism	Progressive	Hypermanganesemia, low serum iron, iron deficiency anemia	IDD	NR	Hyperintensity of the BG and cerebellum	Chelation therapy
*HTT*	AD (CAG repeat expansion)	Childhood to adulthood	Focal	Cervical dystonia, parkinsonism, myoclonus, rigidity, bradykinesia, and chorea	NR	Huntington’s disease	DD/IDD	Learning disabilities, epilepsy, and behavioral and psychiatric manifestations	Nonspecific findings	Symptomatic
*COASY*	AR	Infancy	Focal	Oromandibular dystonia, parkinsonism, spasticity, dysarthria	Progressive	CoPAN, peripheral neuropathy, optic atrophy/pigmentary retinopathy	IDD	Gait impairment, learning difficulties, epilepsy	Iron deposition in GP, Pontocerebellar hypoplasia	NR
*ATP13A2*	AR	Late childhood to adulthood	Focal progressing to generalized	Oromandibular dystonia, parkinsonism spasticity, ataxia, OGC, myoclonus	NR	Kufor Rakeb disease, visual loss	Cognitive deterioration	Epilepsy, ASD, psychosis	Nonspecific findings	Symptomatic

Legend: AD = autosomal dominant; AR = autosomal recessive; ASD = autism spectrum disorder; *ATP13A2* = ATPase cation-transporting 13A2; ATP7B = ATPase copper-transporting beta; BG = basal ganglia; *COASY* = Coenzyme A Synthase; CoPAN = COASY-associated neurodegeneration; DD = developmental delay; GP = globus pallidus; *HTT* = Huntingtin; IDD = intellectual developmental disorder; MD = movement disorder; MRI = magnetic resonance imaging; NR = not reported; OGC = oculogyric crises; *SLC30A10* = solute carrier family 30 member 10; *SLC39A14* = solute carrier family 39 member 14; WD = Wilson disease; XL = X linked.

**Table 6:** Genes associated with complex dystonia with chorea: clinical features, neuroimaging, and treatment options.

Gene	Inheritance	Age at Onset	Dystonia Onset and/or Distribution	Other MD	Course of Dystonia	Other Systemic Features/Associated Diseases	Cognitive Development	Neuropsychiatric Features	Brain MRI	Treatment Option
*HPRT1*	XL	Infancy	Generalized, action dystonia	Opisthotonos, choreoathetosis, occasionally ballism, cerebral palsy	Progressive	LND, delayed growth and puberty	IDD	Self-injurious behavior, oppositional defiance, seizures, gait impairment	Nonspecific findings	Symptomatic
*MMUT*, *MMAA*, *MMAB*, *MMADHC*, *MCEE*	AR	Infancy, childhood, or adolescence	Generalized dystonia	Chorea, spasticity, ataxia	Progressive	Methylmalonic acidemia, acute metabolic crises, hematology alteration,	DD/IDD	Seizures, lethargy, hypotonia	BG injury (GP)	Symptomatic, dietary restriction, L-carnitine
*PCCA*, *PCCB*	AR	Infancy	Focal upper limb dystonia or generalized dystonia after crisis	Choreoathetosis, spasticity	Progressive	Propionic acidemia, acute metabolic crises, ketoacidosis, hyperammonemia, cardiomyopathy, hematology alteration, short stature, gastrointestinal disturbation	DD	Seizure, lethargy, hypotonia,	BG injury (caudate and putamen)	Symptomatic, dietary restriction, L-carnitine
*GCDH*	AR	Infancy	Generalized dystonia	Choreoathetosis, parkinsonism, spasticity, orofacial dyskinesia	Progressive	Glutaric acidemia type 1, acute metabolic crises, macrocephaly, hepatomegaly	IDD	Hypotonia, seizures	BG degeneration, striatal necrosis, frontotemporal atrophy, widening of cortical sulci, symmetrical progressive demyelination	Symptomatic, dietary restriction, L-carnitine
*ETHE1*	AR	Infancy	Generalized dystonia	Chorea, ataxia	Progressive	Ethylmalonic encephalopathy, chronic diarrhea, petechiae, orthostatic acrocyanosis	DD/IDD	Hypotonia, seizures	Hyperintense lesions in the BG	Symptomatic

Legend: AR = autosomal recessive; BG = basal ganglia; DD = developmental delay; GP = globus pallidus; *HPRT1* = hypoxanthine phosphoribosyltransferase 1; IDD: = intellectual developmental disorder; LND = Lesch–Nyhan disease; *MCEE* = Methylmalonyl-CoA epimerase; MD = movement disorder; MRI = magnetic resonance imaging; *MMAA* = metabolism of cobalamin-associated A; *MMAB* = metabolism of cobalamin-associated B; *MMADHC* = metabolism of cobalamin-associated D; *MMUT* = methylmalonyl-CoA mutase; NR = not reported; XL = X linked.

**Table 7:** Genes associated with complex dystonia with epilepsy: clinical features, neuroimaging, and treatment options.

Gene	Inheritance	Age at Onset	Dystonia Onset and/or Distribution	Other MD	Course of Dystonia	Other Systemic Features/Associated Diseases	Cognitive Development	Neuropsychiatric Features	Brain MRI	Treatment Option
*SCN1A*	AD	Infancy	NR	Chorea, ballism, myoclonus, hand stereotypies	Progressive	Dravet Syndrome, dysmorphic features	DD/IDD	Epileptic encephalopathy, inability to speak, and walk	Progressive cortical and WM atrophy, thinning of the CC, impaired myelination	Symptomatic
*UBA5*	AR	Infancy	Generalized and status dystonicus	Spasticity, dystonic, or athetoid movements	Progressive	Delayed growth	DD/IDD	Microcephaly, DEE, axial hypotonia with peripheral hypertonia, inability to speak	Nonspecific findings	Symptomatic
*WWOX*	AR	Early childhood	NR	Spasticity, dyskinetic component, bradykinesia	Progressive	Visual impairment, short stature	DD/IDD	DEE, microcephaly, inability to speak, and walk	Nonspecific findings	Symptomatic
*FOXG1*	AD	Early childhood	Generalized, focal, paroxysmal	Craniofacial involvement, choreoathetosis, stereotypies, akinetic-rigid parkinsonism	Progressive	Rett Syndrome	DD/IDD	Microcephaly, epilepsy, ASD	Nonspecific findings	Symptomatic
*GABRA1*	AD	Infancy	Generalized	Choreoathetosis, spasticity, cerebral palsy	NR	NR	DD/IDD	DEE, seizures, speech impairment, strabismus	Nonspecific findings	Symptomatic
*GRIN1*	AD/AR	Infancy	NR	Mixed MD chorea and dystonia, OGC	NR	Dysmorphic features	DD/IDD	Microcephaly, prominent hypotonia, epilepsy, complex stereotypies, sleep disturbances	Nonspecific findings	Symptomatic
*SPTAN1*	AD	Infancy to early childhood	NR	Ataxia, myoclonus, abnormal ocular movements	NR	NR	DD/IDD	DEE, HSP, ASD	Cerebellar atrophy, delayed myelination, thin CC	Symptomatic
*DNM1*	AD/AR	Childhood	Focal or segmental	Choreoathetosis, spasticity	NR	Visual impairment	Mild to profound DD/IDD	Epilepsy, encephalopathy, ASD, ADHD, aggressive behaviour	Nonspecific findings	Symptomatic

Legend: AD = autosomal dominant; ADHD = attention deficit/hyperactivity disorder; AR = autosomal recessive; ASD = autism spectrum disorder; CC = corpus callosum; DD = developmental delay; DEE = developmental and epileptic encephalopathy; *DNM1* = dynamin 1; *FOXG1* = forkhead box G1; *GABRA1* = gamma-aminobutyric acid type A receptor subunit alpha1; *GRIN1* = glutamate ionotropic receptor NMDA type subunit 1; HSP = hereditary spastic paraplegia; IDD = intellectual developmental disorder; MD = movement disorder; MRI = magnetic resonance imaging; NR = not reported; OGC = oculogyric crises; *SCN1A* = sodium voltage-gated channel alpha subunit 1; *SPTAN1* = spectrin alpha, non-erythrocytic 1; *UBA5* = ubiquitin-like modifier activating enzyme 5; WM = white matter; *WWOX* = WW domain containing oxidoreductase.

**Table 8:** Genes associated with complex dystonia with hearing impairment: clinical features, neuroimaging, and treatment options.

Gene	Inheritance	Age at Onset	Dystonia Onset and/or Distribution	Other MD	Course of Dystonia	Other Systemic Features/Associated Diseases	Cognitive Development	Neuropsychiatric Features	Brain MRI	Treatment Option
*TIMM8A*	XL	Late childhood to adulthood	Focal progressing to generalized	Spasticity, tremor, and postural instability	Progressive	Mohr–Tranebjaerg syndrome, deafness, and visual impairment	Cognitive deterioration	Psychiatric disturbance, dysarthria, and dysphagia	Brain and caudate nuclei atrophy, iron deposition in GP and SN	Symptomatic, DBS
*DCAF17*	AR	Adolescence	Focal progressing to generalized	Dystonic spasms with dystonic posturing, chorea	Progressive	NBIA, Woodhouse–Sakati syndrome, ↓ IGF-1	Mild IDD	Dysarthria and dysphagia	Iron deposition in the GP, SN, and red nucleus	Symptomatic or DBS
*GAMT*, *SERAC1*, *SUCLA2*	AR	Infancy to childhood	Focal/generalized	Ataxia, chorea, athetosis	Progressive	SNHL, deafness, failure to thrive, and early death	IDD	Hyperactivity, ASD, self-injurious behavior, seizures, speech disorder	Pathologic intensities in the BG, hypomyelination of WM	Symptomatic
*SLC6A8*, *BCAP31*	XL									
*ACTB*	AD	Adolescence	Focal progressing to generalized	Bulbar dysfunction	Progressive	Congenital deafness, skeletal abnormalities	DD/IDD	Dysarthria and dysphagia	Nonspecific findings	Symptomatic, DBS

Legend: ↓ = low; *ACTB* = actin beta; AD = autosomal dominant; AR = autosomal recessive; ASD = autism spectrum disorder; *BCAP31* = B cell receptor-associated protein 31; BG = basal ganglia; DBS = deep brain stimulation; *DCAF17* = DDB1 and CUL4-associated factor 17; DD = developmental delay; *GAMT* = guanidinoacetate N-methyltransferase; GP = globus pallidus; IGF-1 = insulin-like growth factor-1; IDD = intellectual developmental disorder; MD = movement disorder; MRI = magnetic resonance imaging; NBIA = neurodegeneration with brain iron accumulation; *SERAC1* = serine active site containing 1; *SLC6A8* = solute carrier family 6 member 8; SN = substantia nigra; *SNHL* = sensorineural hearing loss; *SUCLA2* = succinate–CoA ligase ADP-forming subunit beta; *TIMM8A* = translocase of inner mitochondrial membrane 8A; XL = X linked; WM = white matter.

**Table 9:** Genes associated with complex dystonia with ocular impairment: clinical features, neuroimaging, and treatment options.

Gene	Inheritance	Age at Onset	Dystonia Onset and/or Distribution	Other MD	Course of Dystonia	Other Systemic Features/Associated Diseases	Cognitive Development	Neuropsychiatric Features	Brain MRI	Treatment Option
*POLG*	AR/AD	Adolescence to adulthood	Focal/generalized	Ataxia, myoclonus, choreoathetosis, parkinsonism	Progressive	External ophthalmoplegia, liver, gastrointestinal, and renal diseases, hearing loss	DD	Seizures, dysarthria, mood disorder, sleep disorders	Nonspecific findings	Symptomatic
*SLC19A3*	AR	Childhood	Generalized	Ataxia, rigidity	Progressive	External ophthalmoplegia, quadriparesis, coma, and death	DD/IDD	Acute encephalopathy, dysarthria, dysphagia, loss of developmental milestones, and seizures	Symmetric and bilateral necrosis in the BG with severe edema	Thiamine and/or biotin therapy
*MECR*	AR	Infancy to childhood	Generalized	NR	Progressive	Optic atrophy	Normal	Epilepsy and dysarthria	Symmetric and bilateral BG abnormalities	Symptomatic, DBS

Legend: AD = autosomal dominant; AR = autosomal recessive; BG = basal ganglia; DBS = deep brain stimulation; DD = developmental delay; IDD = intellectual developmental disorder; MD = movement disorder; *MECR* = mitochondrial trans-2-enoyl-CoA reductase; MRI = magnetic resonance imaging; NR = not reported; *POLG* = DNA polymerase gamma, catalytic subunit; *SLC19A3* = solute carrier family 19 member 3.

**Table 10:** Genes associated with paroxysmal dyskinesias: clinical features, neuroimaging, and treatment options.

Gene	Inheritance	Age at Onset	Dystonia/Dyskinesia Features	Type of PxMD	Cognitive Development	Neuropsychiatric Features	Brain MRI	Treatment Option
*PRRT2*	AD	Childhood, adolescence	Dystonic postures, chorea, or athetosis	PKD	Normal, mild DD/IDD	Epilepsy, anxiety, depression, sleep disorders, ADHD	Normal	SCB
*TMEM151A*	AD	Childhood, adolescence	Dystonia with facial involvement	PKD	NR	Occasional migraine or tremor, epilepsy	Normal	SCB
*RHOBTB2*	AD	Infancy, childhood	Choreodystonia, ataxia, and/or stereotypies	PKD	Severe DD/IDD	ASD	Normal or unspecific findings	SCB
*SCN8A*	AD	Childhood	Orobuccolingual dyskinesia, choreiform movements, tremor	PKD	DD/IDD	Epilepsy, ASD, ADHD	Unspecific findings	SCB, KD
*SLC2A1*	AD	Childhood	Foot dystonia, paroxysmal choreoathetosis, progressive spastic paraplegia	PED	DD/IDD	Epilepsy	Normal	KD, triheptanoin
*TBC1D24*	AR	Childhood	Dystonic movements, myoclonus, ataxia, dysmetria, dysarthria	PED	Normal, DD/IDD	Epilepsy	Non-progressive pontocerebellar hypoplasia	SCB, Acetazolamide
*ECHS1*	AR	Childhood	Generalized dystonia, hemydystonia, focal limb dystonia, torticollis	PED	IDD	Leigh-like syndrome	Bilateral hyperintensity of the GP	Dietary protein restriction and symptomatic supplementation
*PNKD*	AD	Childhood, early adolescence	Dystonia, chorea, athetosis	PNKD	Normal	Tourette syndrome, Tic disorder, anxiety, depression	Normal	BZD
*KCNMA1*	AD	Infancy, early childhood	Dystonic movements and ocular signs	PNKD	DD/IDD	Epilepsy	Normal	Symptomatic

Legend: AD = autosomal dominant; ADHD = attention deficit hyperactivity disorder; AR = autosomal recessive; ASD = autism spectrum disorder; BZD = benzodiazepines; DD = developmental delay; IDD = intellectual developmental disorder; *ECHS1* = enoyl-CoA hydratase, short chain 1; GP = globus pallidus; *KCNMA1* = potassium calcium-activated channel subfamily M alpha 1; KD = ketogenic diet; MRI = magnetic resonance imaging; NR = not reported; PED = paroxysmal exercise/exertion-induced dyskinesia; PKD = paroxysmal kinesigenic dyskinesia; PNKD = paroxysmal non-kinesigenic dyskinesia; *PNKD* = PNKD metallo-beta-lactamase domain containing; *PRRT2* = proline-rich transmembrane protein 2; PxMD = paroxysmal movement disorder; *RHOBTB2* = rho-related BTB domain containing 2; SCB = sodium channel blockers; *SLC2A1* = solute carrier family 2 member 1; *SCN8A* = sodium voltage-gated channel alpha subunit 8; *TBC1D24* = TBC1 domain family member 24; *TMEM151A* = transmembrane protein 151A.

## Data Availability

No new data were created or analyzed in this study. Data sharing is not applicable to this article.

## Abbreviations

AADC: Aromatic L-amino acid decarboxylase; ACMG-AMP: American College of Medical Genetics and Genomics–Association for Molecular Pathology; AD: Autosomal dominant; ADHD: Attention deficit hyperactivity disorder; AGS: Aicardi–Goutières Syndrome; ANAD: Atypical neuroaxonal dystrophy; AR: Autosomal recessive; ASD: Autism spectrum disorder; ASMs: Antiseizure medications; ATP: Adenosine triphosphate; BG: Basal ganglia; BT: Botulinum toxin; BZD: Benzodiazepines; cAMP: Cyclic adenosine monophosphate; CAPOS: Cerebellar ataxia, peripheral neuropathy, optic atrophy, and sensorineural hearing loss; Corpus callosum; CHOR: Chorea; DBS: Deep brain stimulation; CoPAN: COASY-associated neurodegeneration; DD: Developmental delay; DDS: Deafness–dystonia syndromes; DEEs: Developmental and epileptic encephalopathies; DOORS: Deafness, onychodystrophy, osteodystrophy, impaired intellectual development, and seizures syndrome; DYT: Dystonia; DTI: Diffusion tensor imaging; eIF2α: Eukaryotic initiation factor 2 alpha; EMA: European Medicines Agency; FAHN: Fatty acid hydroxylase-associated neurodegeneration; FDA: Food and Drug Administration; GP: Globus pallidum; GPi: Internal globus pallidum; GPi-DBS: Deep brain stimulation of the globus pallidus internus; Gαolf: α subunit of the stimulatory G protein; H-ABC: Hypomyelination with atrophy of the basal ganglia and cerebellum; HD: Huntington’s disease; HOPS: Homotypic fusion and vacuole protein sorting; HPRT: Hypoxanthine-guanine phosphoribosyl transferase; HSP: Hereditary spastic paraplegia; H3K4: Fourth lysine of histone H3; IDD: Intellectual developmental disorder; IEM: Inborn errors of metabolism; IGF-1: Insulin-like growth factor-1; INAD: Infantile neuroaxonal dystrophy; KCTD: Potassium channel tetramerization domain; KD: Ketogenic diet; K4: Fourth lysine; LND: Lesch–Nyhan disease; LSDs: Lysosomal storage disorders; MEGDEL: 3-methylglutaconic aciduria with deafness–encephalopathy–Leigh-like; MD: Movement disorder; MELAS: Mitochondrial encephalopathy, lactic acidosis, and stroke-like episodes; MIDs: Mitochondrial inherited disorders; MPAN: Mitochondrial membrane protein-associated neurodegeneration; MRI: Magnetic resonance imaging; NBIA: Neurodegeneration with brain iron accumulation; NAD: Neuroaxonal dystrophy; NEMMLAS: Neurodevelopmental mitochondrial disorder with abnormal movements and lactic acidosis with or without seizures; NMDA: N-methyl-D-aspartate; NPC: Niemann–Pick disease type C; NR: Not reported; OADs: Organic acidurias; OCD: Obsessive–compulsive disorder; ODD: Oppositional defiant disorder; OGC: Oculogyric crises; PARK: Parkinsonism; PED: Paroxysmal exercise/exertion-induced dyskinesia; Phe = Phenylalanine; PKAN: Pantothenate kinase-associated neurodegeneration; PKD: Paroxysmal kinesigenic dyskinesia; PKDYS1: Infantile-onset parkinsonism-dystonia-1; PNKD: Paroxysmal nonkinesigenic dyskinesia; PRKRA: Protein activator of the interferon-induced protein kinase; PxMD: Paroxysmal movement disorders; SCB: Sodium channel blockers; SD: Status dystonicus; SN: Substantia nigra; SNDC: Childhood-onset striatonigral degeneration; SNHL: Sensorineural hearing loss; SPG35: Spastic paraplegia-35; WD: Wilson’s disease; WM: white matter; XL: X-linked.
